# Supplementary material for: Calibrating a network meta-analysis of diabetes trials of sodium glucose cotransporter 2 inhibitors, glucagon-like peptide-1 receptor analogues and dipeptidyl peptidase-4 inhibitors to a representative routine population: a systematic review protocol
Source: BMJ Open. 2022 Oct 27;12(10):e066491. doi: 10.1136/bmjopen-2022-066491 (PMC9621152; doi:10.1136/bmjopen-2022-066491)
Supplement: Supplementary data [file bmjopen-2022-066491supp001.pdf]

Supplementary appendix

Section 1: Search strategies

Medline database

Table S1: Medline search strategy

# ▲	Searches	Results
1	non-insulin dependent diabetes mellitus/	125129
2	glucose intolerance/	8257
3	diabetic obesity/	0
4	impaired glucose tolerance/	8257
5	(non-insulin* depend* or noninsulin* depend* or noninsulindepend* or non insulindepend*).tw.	12075
6	((typ* 2 or typ* II) adj4 diabet*).tw.	132687
7	((adult* or matur* or late or slow or stabl* or obes*) adj4 diabet*).tw.	53350
8	(T2D* or DM2 or IIDM or MODY or NIDDM).tw.	35508
9	((nonketo* or non keto* or ketoresist* or keto resist*) adj4 diabet*).tw.	491
10	impaired glucose toleran*.tw.	10516
11	glucose intoleran*.tw.	10077
12	insulin* resistan*.tw.	76852
13	(insulin* defic* adj2 relativ*).tw.	184
14	(metabolic* syndrom* or plurimetabolic* syndrom*).tw.	46774
15	glucagon like peptide 1 receptor agonist/	0
16	(glucagon-like peptide 1 receptor inhibitor* or glucagon-like peptide 1 receptor agonist* or glucagon-like peptide 1 inhibitor* or glucagon-like peptide 1 agonist* or GLP-1 receptor inhibitor* or GLP-1 receptor agonist* or GLP-1 inhibitor* or GLP-1 agonist*).tw.	3107
17	albiglutide/	0
18	dulaglutide/	0
19	exendin 4/	2290
20	liraglutide/	1479
21	lixisenatide/	0
22	semaglutide/	0
23	taspoglutide/	0
24	albiglutide.tw.	168

Butterly E, et al. BMJ Open 2022; 12:e066491. doi: 10.1136/bmjopen-2022-066491

25	dulaglutide.tw.	260
26	(exenatide or exendin 4).tw.	3063
27	liraglutide.tw.	2254
28	lixisenatide.tw.	342
29	semaglutide.tw.	254
30	taspoglutide.tw.	56
31	dipeptidyl peptidase IV inhibitor/	3609
32	(dipeptidyl-peptidase IV Inhibitor* or dipeptidyl-peptidase 4 Inhibitor* or ((DPP4 or DPP 4 or DPP IV) adj inhibitor*)).tw.	4570
33	alogliptin/	0
34	anagliptin/	0
35	gemigliptin/	0
36	linagliptin/	369
37	omarigliptin/	0
38	saxagliptin/	0
39	sitagliptin/	1300
40	teneligliptin/	0
41	vildagliptin/	592
42	alogliptin.tw.	419
43	anagliptin.tw.	59
44	gemigliptin.tw.	49
45	linagliptin.tw.	615
46	omarigliptin.tw.	37
47	saxagliptin.tw.	594
48	sitagliptin.tw.	2029
49	teneligliptin.tw.	116
50	vildagliptin.tw.	904
51	evogliptin.tw.	19
52	evogliptin/	0
53	sodium glucose cotransporter 2 inhibitor/	0
54	(sodium glucose transporter 2 inhibitor* or sodium glucose transporter ii inhibitor* or SGLT 2 inhibitor*).tw.	454
55	(sodium glucose cotransporter adj3 inhibitor*).tw.	1192
56	(sodium glucose co transporter adj3 inhibitor*).tw.	719
57	canagliflozin/	515
58	dapagliflozin/	0
59	empagliflozin/	0
60	ertugliflozin/	0
61	tofogliflozin/	0
62	canagliflozin.tw.	789
63	dapagliflozin.tw.	818
64	empagliflozin.tw.	862
65	ertugliflozin.tw.	61

66	tofogliflozin.tw.	86
67	ipragliflozin/	0
68	ipragliflozin.tw.	166
69	Randomized Controlled Trials as Topic/	126623
70	randomized controlled trial/	490217
71	Random Allocation/	100528
72	Double Blind Method/	153484
73	Single Blind Method/	27365
74	clinical trial/	518141
75	clinical trial, phase i.pt.	19368
76	clinical trial, phase ii.pt.	31271
77	clinical trial, phase iii.pt.	15567
78	clinical trial, phase iv.pt.	1754
79	controlled clinical trial.pt.	93274
80	randomized controlled trial.pt.	490217
81	multicenter study.pt.	257365
82	clinical trial.pt.	518141
83	exp Clinical Trials as topic/	330616
84	(clinical adj trial$).tw.	334395
85	((singl$ or doubl$ or treb$ or tripl$) adj (blind$3 or mask$3)).tw.	164050
86	PLACEBOS/	34468
87	placebo$.tw.	204278
88	randomly allocated.tw.	26477
89	(allocated adj2 random$).tw.	29629
90	or/69-89	1553327
91	or/1-14	295318
92	or/15-30	6907
93	or/31-52	7002
94	or/53-68	3313
95	92 or 93 or 94	15069
96	90 and 91 and 95	4292
97	case report.tw.	289645
98	letter/	1036792
99	historical article/	354203
100	or/97-99	1665591
101	96 not 100	4237
102	limit 101 to human	3612
103	limit 102 to english language	3452
104	limit 103 to yr="2002-Current"	3452

Embase database

Table S2: Embase search strategy

▲	**Searches**	**Results**
1	((diabetes or diabetes mellitus or diabetic*) adj1 (type 2 or type II or type ii or non-insulin dependent or noninsulin dependent or adult onset or mature onset or late onset)).tw	219042
2	(diabetic nephropath* or diabetic kidney disease).tw.	26837
3	glucose intolerance/	17965
4	diabetic obesity/	3737
5	impaired glucose tolerance/	30222
6	(non insulin* depend* or noninsulin* depend* or noninsulindepend* or non insulindepend*).tw.	14344
7	((typ* 2 or typ* II) adj4 diabet*).tw.	213585
8	((adult* or matur* or late or slow or stabl* or obes*) adj4 diabet*).tw.	85106
9	(T2D* or DM2 or IIDM or MODY or NIDDM).tw.	64908
10	((nonketo* or non keto* or ketoresist* or keto resist*) adj4 diabet*).tw.	721
11	impaired glucose toleran*.tw.	16435
12	glucose intoleran*.tw.	15372
13	insulin* resistan*.tw.	116387
14	(insulin* defic* adj2 relativ*).tw.	310
15	(metabolic* syndrom* or plurimetabolic* syndrom*).tw.	76726
16	glucagon like peptide 1 receptor agonist/	4508
17	(glucagon-like peptide 1 receptor inhibitor* or glucagon-like peptide 1 receptor agonist* or glucagon-like peptide 1 inhibitor* or glucagon-like peptide 1 agonist* or<br>GLP-1 receptor inhibitor* or GLP-1 receptor agonist* or GLP-1 inhibitor* or GLP-1 agonist*).tw.	5749
18	albiglutide/	871
19	dulaglutide/	1104
20	exendin 4/	9969
21	liraglutide/	7911
22	lixisenatide/	1317
23	semaglutide/	861
24	taspoglutide/	254
25	albiglutide.tw.	332
26	dulaglutide.tw.	654
27	(exenatide or exendin 4).tw.	5902
28	liraglutide.tw.	4839
29	lixisenatide.tw.	693
30	semaglutide.tw.	492
31	taspoglutide.tw.	110
32	dipeptidyl peptidase IV inhibitor/	8745
33	(dipeptidyl-peptidase IV Inhibitor* or dipeptidyl-peptidase 4 Inhibitor* or ((DPP4 or<br>DPP 4 or DPP IV) adj inhibitor*)).tw.	8196

Butterly E, et al. BMJ Open 2022; 12:e066491. doi: 10.1136/bmjopen-2022-066491

34	alogliptin/	1740
35	anagliptin/	198
36	gemigliptin/	171
37	linagliptin/	2337
38	omarigliptin/	116
39	saxagliptin/	2941
40	sitagliptin/	7976
41	teneligliptin/	338
42	vildagliptin/	3759
43	alogliptin.tw.	739
44	anagliptin.tw.	122
45	gemigliptin.tw.	110
46	linagliptin.tw.	1304
47	omarigliptin.tw.	59
48	saxagliptin.tw.	1226
49	sitagliptin.tw.	4148
50	teneligliptin.tw.	238
51	vildagliptin.tw.	1744
52	evogliptin.tw.	37
53	evogliptin/	48
54	sodium glucose cotransporter 2 inhibitor/	3435
55	(sodium glucose transporter 2 inhibitor* or sodium glucose transporter ii inhibitor* or SGLT 2 inhibitor*).tw.	933
56	(sodium glucose cotransporter adj3 inhibitor*).tw.	1827
57	(sodium glucose co transporter adj3 inhibitor*).tw.	1295
58	canagliflozin/	2568
59	dapagliflozin/	2975
60	empagliflozin/	2877
61	ertugliflozin/	307
62	tofogliflozin/	286
63	canagliflozin.tw.	1536
64	dapagliflozin.tw.	1926
65	empagliflozin.tw.	1803
66	ertugliflozin.tw.	143
67	tofogliflozin.tw.	166
68	ipragliflozin/	484
69	ipragliflozin.tw.	284
70	or/1-15	455561
71	or/16-31	19351
72	or/32-53	19026
73	or/54-69	9181
74	71 or 72 or 73	38216
75	Clinical Trial/	984789
76	Randomized Controlled Trial/	576557

Butterly E, *et al. BMJ Open* 2022; 12:e066491. doi: 10.1136/bmjopen-2022-066491

77	controlled clinical trial/	465986
78	multicenter study/	231443
79	Phase 3 clinical trial/	43062
80	Phase 4 clinical trial/	3641
81	exp RANDOMIZATION/	85032
82	Single Blind Procedure/	36861
83	Double Blind Procedure/	169262
84	Crossover Procedure/	61406
85	PLACEBO/	353947
86	randomi?ed controlled trial$.tw.	213219
87	rct.tw.	34296
88	(random$ adj2 allocat$).tw.	41310
89	single blind$.tw.	23926
90	double blind$.tw.	209043
91	((treble or triple) adj blind$).tw.	1070
92	placebo$.tw.	303630
93	Prospective Study/	557933
94	or/75-93	2246792
95	Case Study/	73901
96	case report.tw.	420554
97	abstract report/ or letter/	1124332
98	Conference proceeding.pt.	0
99	Conference abstract.pt.	3581657
100	Editorial.pt.	633720
101	Letter.pt.	1089718
102	Note.pt.	774711
103	or/95-102	6547596
104	94 not 103	1690232
105	70 and 74 and 104	6157
106	limit 105 to human	6042
107	limit 106 to english language	5816
108	limit 107 to yr="2002 -Current"	5812

Butterly E, *et al. BMJ Open* 2022; 12:e066491. doi: 10.1136/bmjopen-2022-066491

Section 2: Protocol for routine healthcare data target population

Scope:

The scope of this document is to set out a protocol for identifying a clinically appropriate target population for calibration modelling within the routine datasets.

Aim:

1) To identify a clinically appropriate target population within the Scottish diabetes register for calibration modelling of a large network meta-analysis of glucose lowering drugs
2) Document the variables to be collected and summarised within the identified population

Background

For the proposed calibration modelling to be clinically relevant, the routine data target population to which the models are applied requires to be clearly set out and clinically justifiable. Using a 2019 extract of the SCI-diabetes database we aim to identify a population of people with type 2 diabetes mellitus where prescription of any of the three drug classes of interest (Sodium Glucose Co-Transporter 2 Inhibitors (SGLT2i) /Glucagon-Like Peptide 1 Receptor Agonists (GLP1ra) / Dipeptidyl Peptidase-4 Inhibitors (DPP4i)) would realistically be considered should the individual require treatment escalation. We aim to exclude anyone who would be considered to have a significant contraindication to any of the three drug classes.

Subsequent work will include clustering to identify more specific subsets of the population e.g., based on age, sex, body weight, renal function, cardiovascular risk. This will allow calibration to more specific subsets of the overall target population. This will be described in a later document.

Pilot work

We conducted some exploratory searches of the 2017 extract of SCI-diabetes to help guide this protocol. We identified those within the register who were prescribed at least one of the drug classes of interest. Overall, we identified 56,867 people on at least one of the target drugs. (Mean age 64.65 years, weight 98.14kg, glycated haemoglobin (HbA1c) 66.97mmol/mol, systolic blood pressure 136.55mmHg, estimated glomerular filtration rate (eGFR) 58.45. ml/min/1.73m^2).

Proposed steps

1) Access the 2019 data extract- and familiarise with datasets available within and data included in each.
2) Limit included participants to those where absolute contraindications for proposed drug classes are absent (see specific exclusions).
3) Extract data on descriptive variables from Table 1 where available.
4) Continuous observations for each individual will be taken as the mean of measurements over 3 years prior to 1/1/19. The most recent measurement in last 3 years will be taken for categorical variables e.g. smoking.
5) If all of the following variables are missing for the last 3 years, we will presume likely that the individual has either moved away or is not engaged with clinical services and they will not be included: HbA1c, systolic blood pressure, diastolic blood pressure, smoking status, fasting plasma glucose, urinary albumin creatinine ratio, total cholesterol, high density lipoprotein cholesterol, low density lipoprotein cholesterol, eGFR and body mass index.
6) Previous comorbidities/prescriptions will be extracted as present if appear in previous 10 years of data.
7) Comorbidity data will be defined using ICD10 codes within the linked SMR01 dataset (specified below). As per large cardiovascular outcome trials e.g. CANVAS[1], history of

cardiovascular disease will be defined as history of atherosclerotic cardiovascular disease including coronary, cerebrovascular or peripheral vascular disease.

8) Comorbidity data from SMR01/prescribing data will be included where the comorbidity appears in any position in the discharge data e.g., primary diagnosis or any other position of diagnosis
9) Create preliminary definition of overall population to be used for calibration based on above which may include modification of variables collected based on availability.
10) Provide summary statistics including number included/excluded to steering committee and (PRIOR to performing calibration or running NMA model on trial data) amend target population protocol on basis of feedback.

Proposed population defining characteristics

Timeframe:

- Date of diagnosis of type 2 diabetes mellitus at least one year prior to 1st Jan 2019
- Limit comorbidity data to a ten year look back
- Must be alive at time of extraction therefore exclude if death on or before chosen date (1st January 2019)

Age:

- Limit to 18 years old or above on 1st Jan 2019 or at diagnosis of diabetes
- No upper limits based on current age or age at diagnosis

Sex:

- No limits based on sex

Diagnosis:

- Must have documented diagnosis of type 2 diabetes mellitus within the derived diagnosis variable in dataset diagnosed before or on 1/1/18
- There will be no limits to the duration of diabetes diagnosis
- Those with diabetes in remission will be excluded when HbA1c limit applied.

Glycaemic control:

- No limit to HbA1c at diagnosis
- Limit population to those with most recent HbA1c ≥53mmol/mol or those with HbA1c <53mmol/mol but currently on one of the three drug classes of interest, or insulin.

Body Mass Index:

- Limit to those with most recent BMI measurement to ≥23.5kg/m^2 (use cleaned variable either from clinician entered variable from Sci Diabetes, or derived from weight/height)
- More specific BMI groupings will likely be considered within the clustering subsets.
- Provide summary data to the steering committee regarding those who would be excluded should the BMI cutoff be changed to 20 or 25 kg/m^2
-

Current drugs:

- It will be permissible for those within the target population to be on one or two of the three target drug classes as excluding these people is likely to unfavourably skew the target population.
- It will also be permissible to be taking other glucose lowering drugs including insulin, metformin, sulfonylureas, thiazolidinediones, alpha glucosidase inhibitors.
- There will be no limits on non-diabetes drugs including antihypertensives, ACEi/ARB, statins or antiplatelets
- There will be a limit on high dose oral steroids- exclude if currently on ≥prednisolone 5mg or equivalent (BNF codes 1.5.2, 6.3.2, 10.1.2) as of 1/1/19

Renal function:

- Limit to those with eGFR >30 ml/min/1.73m^2 (derived CKD EPI variable from within Diabepi).
- Exclude if current renal replacement therapy (linked Renal Registry Data within Diabepi)

Cardiovascular disease/risk:

- There will be no limit on prior cardiovascular disease, including heart failure, or cardiovascular risk factors e.g., smoking, dyslipidaemia at this stage.
- These factors will be considered further in the subset clustering
- ICD 10 codes for CV disease include coronary disease, cerebrovascular ischaemic disease, unspecified cerebral infarction, unspecified atherosclerosis, and peripheral vascular disease. (I have excluded haemorrhagic stroke disease when specified)

Specific exclusions:

- Any type of diagnosed diabetes other than type 2 diabetes mellitus
- Admission with DKA in the last 10 years defined via ICD10 codes linked to SMR01 admission data (ICD10: E10.1, E11.1, E13.1, E14.1) Note E10.1 is type 1 with ketoacidosis but leave in as check in case of coding errors.
- Renal function: Most recent eGFR≤ 30 ml/min/1.73m^2
- Urinary tract infection: Exclude if hospitalisation for urinary tract infection/urinary sepsis in last 10 years defined via ICD10 codes linked to SMR01 admission data (IC10: N39.0)
- Fungal infections: Exclude if 3 or more prescriptions for anti-fungal medication (oral, pessary or topical >1% strength) within the preceding 3 years defined using BNF code 5.2 within linked prescribing data. Whilst this will not 100% identify genitourinary fungal infections vs other dermatological fungal infections, limiting to oral, pessary and higher strength topical treatments is likely to limit the overlap somewhat.
- Pancreatitis/Pancreatic Insufficiency: Exclude if previously admitted to hospital with pancreatitis or pancreatic insufficiency in last 10 years defined via ICD10 codes linked to SMR01 admission data (ICD10: K85.0, K85.1, K85.2, K85.3, K85.8, K85.9, K86.0, K86.1, K87.1, (B25.2, B26.3)) or prescription of Pancreatin/Creon supplements as of 1/1/19 defined by BNF code 1.9.4
- Gallstone disease: Exclude if hospitalised with cholelithiasis or cholecystitis disease in last 10 years defined via ICD10 codes linked to SMR01 admission data (ICD10: K80.0, K80.1, K80.2, K80.3, K80.4, K80.5, K80.8, K81.0, K81.1, K81.8, K81.9). If person has had subsequent cholecystectomy can be included (OPCS surgical codes J18.1, J18.2, J18.3, J18.4, J18.5, J18.8, J18.9).
- Inflammatory Bowel Disease: Exclude if hospital admission with inflammatory bowel disease (UC/Crohn's Disease/Unspecified non infective inflammatory bowel disease) in last 10 years

defined via ICD10 codes linked to SMR01 admission data (ICD 10: K50.0 , K50.1, K50.8, K50.9, K51.0, K51.2, K51.3, K51.4, K51.5, K51.8, K51.9 , K52.0, K52.1, K52.2, K52.3, K52.8, K52.9) Also exclude if immunotherapy (Unable to find with 1.5.3 BNF code. Instead used drugnames from non-steroid drugs mentioned in "British Society of Gastroenterology consensus guidelines on the management of inflammatory bowel disease in adults" = MESALAZINE', 'AZATHIOPRINE', 'MERCAPTOPURINE', 'METHOTREXATE','INFLIXIMAB', 'ADALIMUMAB', 'GOLIMUMAB', 'VEDOLIZUMAB', 'TOFACITINIB', 'USTEKINUMAB') plus ≥ 1 outpatient appointment at Gastroenterology within past 3 years based on SMR00 coding (Specialty= A9, attendance status= 1 (seen)).
Whilst this will not identify those with milder disease in the community, and may include people with other diagnoses in error, in practical terms it will likely identify and exclude those with more severe disease in whom incretin therapies would be contraindicated.

- Gastroparesis: Whilst we intended to exclude for history of gastroparesis, there is no ICD10 code specific enough for this therefore it was not possible on the available data.
- Recent diagnosis of cancer (based on record in the smr06 cancer register database in last 3 years).
- End of Life: Exclude, based on SMR01 data, if admission from or discharge to a hospice at any time (location code =62), admission under palliative care (spec=AM), admission reason palliative care or geriatric palliative care (admreas=1M/4B) or admission to palliative care facility (sigfac=1G), as treatment unlikely to be appropriate

Variables of interest within target population

Aggregate descriptive characteristics from the target population will be gathered to facilitate trial outcome calibration in the next stage of this project.

Table S1: Variables of interest within routine datasets

1. Age in years
2. Duration of diabetes in years
3. Sex
4. BMI in kg/m^2
5. Ethnicity/Race
6. Systolic blood pressure in mmHg
7. Diastolic blood pressure in mmHg
8. Smoking status (never, previously, currently)
9. Previous cardiovascular disease (ICD10: I20.0, I20.1, I20.8, I20.9, I21.0, I21.1, I21.2, I21.3, I21.4, I21.9, I22.0, I22.1, I22.8, I22.9, I23.0, I23.1, I23.2, I23.3, I23.4, I23.5, I23.6, I23.8, I24.0 I24.1, I24.8, I24.9, I25.0, I25.1, I25.2, I25.3, I25.4, I25.5, I25.6, I25.8, I25.9, I63.0, I63.1, I63.2, I63.3, I63.4, I63.5, I63.6, I63.8, I63.9, I64.0, I65.0 , I65.1, I65.2, I65.3, I65.8, I65.9, I66.0, I66.1, I66.2, I66.3, I66.4, I66.8, I66.9, I67.2, I67.8, I67.9, I69.3, I69.4, I69.8, I70.0, I70.1, I70.2, I70.8, I70.9 , I73.0, I73.1, I73.8, I73.9) (yes or no)
10. History of heart failure (ICD: I50.0, I50.1, I50.9 in SMR01 data and/or currently on furosemide or bumetanide) (yes or no)
11. Current non-insulin glucose lowering agents (BNF codes: 6.1.2) (yes/no)
12. Current Insulin (yes or no) (BNF codes: 6.1.1)
13. HbA1c (mean of recent) in mmol/mol

14.	Mean of recent eGFR ml/min/1.73m^2 (CKD EPI)
15.	Urine albumin to creatinine ratio in mg/g
16.	Total cholesterol in mmol/l
17.	Low Density Lipoprotein (LDL) in mmol/l
18.	High Density Lipoprotein (HDL) in mmol/l

Section 3: Statistical methods

Models will be fitted using the multilevel network meta-regression framework described by Phillippo et al[2], which we outline here.

IPD studies provide outcomes y_{ijk} and a vector of covariates $\boldsymbol{x}_{ijk}$ for each individual i in study j receiving treatment k. The individual-level model for these data is:

$$y_{ijk} \sim \pi_{\text{Ind}}(\theta_{ijk})$$
$$g(\theta_{ijk}) = \eta_{jk}(\boldsymbol{x}_{ijk}) = \mu_j + \boldsymbol{x}_{ijk}^T(\boldsymbol{\beta}_1 + \boldsymbol{\beta}_{2,k}) + \gamma_k$$

where $\pi_{\text{Ind}}(\cdot)$ is a suitable likelihood distribution. $g(\cdot)$ a suitable link function, which transforms the expected outcome θ_{ijk} for an individual conditional on their covariates onto the linear predictor η_{ijk}. μ_j are study-specific intercepts, $\boldsymbol{\beta}_1$ and $\boldsymbol{\beta}_{2,k}$ correspond to the effects of covariates and covariate-treatment interactions respectively, and γ_k is the individual-level treatment effect of treatment k compared to a chosen network reference treatment 1.

Aggregate studies provide aggregate outcomes $y_{\bullet jk}$ on treatment k in study j, and a joint distribution for the covariates $f_{jk}(\boldsymbol{x})$. The aggregate-level model for these data is constructed by integrating the individual-level model over the population in each study:

$$y_{\bullet jk} \sim \pi_{\text{Agg}}(\theta_{\bullet jk})$$
$$\theta_{\bullet jk} = \int_{\mathfrak{X}} g^{-1}\left(\eta_{jk}(\boldsymbol{x})\right) f_{jk}(\boldsymbol{x})\, d\boldsymbol{x}$$

where $\pi_{\text{Agg}}(\cdot)$ is a suitable likelihood distribution, $\theta_{\bullet jk}$ is the expected outcome on treatment k in study j, and $\mathfrak{X}$ is the support of the covariates. The integral is evaluated using efficient quasi-Monte Carlo numerical integration, with a sample of S points $\tilde{\boldsymbol{x}}_{jk;s}$ from the joint distribution $f_{jk}(\boldsymbol{x})$:

$$\theta_{\bullet jk} \approx S^{-1} \sum_{\tilde{\boldsymbol{x}}_{jk;s}} g^{-1}\left(\eta_{jk}(\tilde{\boldsymbol{x}}_{jk;s})\right).$$

The joint distribution of covariates $f_{jk}(\boldsymbol{x})$ is rarely available directly from study publications; instead, marginal summaries are available (e.g. means and standard deviations, proportions). However, under assumptions about the forms of the marginal distributions and the correlation structure (for example based on those observed in the IPD studies), the full joint distribution can be reconstructed. In practice, results are seen to be robust to misspecification of these assumptions[3].

In a Bayesian framework, prior distributions will be placed on each of the model parameters μ_j, $\boldsymbol{\beta}_1$, $\boldsymbol{\beta}_{2,k}$, γ_k. Random effects models and unrelated mean effects or node-splitting models will also be fitted within the above framework, to explore heterogeneity and inconsistency respectively.[2]

After model fitting, population-average treatment effects $d_{ab(P)}$ between any two treatments a and b, in a population P with mean covariate values $\overline{\boldsymbol{x}}_{(P)}$, can be obtained as

$$d_{ab(P)} = \bar{\boldsymbol{x}}_{(P)}^{T}\left(\boldsymbol{\beta}_{2,b} - \boldsymbol{\beta}_{2,a}\right) + \gamma_b - \gamma_a.$$

When outcomes are reported by subgroup in the aggregate studies, these can be incorporated by extending the aggregate-level model above as follows:

$$y_{\bullet jkl} = \pi_{\text{Agg}}\left(\theta_{\bullet jkl}\right)$$

$$\theta_{\bullet jkl} \approx S_{jkl}^{-1} \sum_{\tilde{\boldsymbol{x}}_{jkl;s}} g^{-1}\left(\eta_{jk}\left(\tilde{\boldsymbol{x}}_{jkl;s}\right)\right)$$

where for each subgroup l the integration points from the full joint distribution are partitioned into each subgroup as $\tilde{\boldsymbol{x}}_{jkl;s}$. This approach is only appropriate for independent subgroups (e.g. levels of a single covariate, or subgroups of multiple covariates reported factorially). For non-independent subgroups (e.g. multiple single-covariate subgroup analyses), this approach will be extended to account for the resulting correlations in the likelihood.

References

1. Neal B, Perkovic V, de Zeeuw D, Mahaffey KW, Fulcher G, Stein P, et al. Rationale, design, and baseline characteristics of the Canagliflozin Cardiovascular Assessment Study (CANVAS)—A randomized placebo-controlled trial. Am Heart J. 2013 Aug 1;166(2):217-223.e11.

2. Phillippo DM, Dias S, Ades AE, Belger M, Brnabic A, Schacht A, et al. Multilevel network meta-regression for population-adjusted treatment comparisons. J R Stat Soc Ser A Stat Soc. 2020;183(3):1189–210.

3. Phillippo DM, Dias S, Ades AE, Welton NJ. Assessing the performance of population adjustment methods for anchored indirect comparisons: A simulation study. Stat Med. 2020;39(30):4885–911.
