# Supplementary material for: Calibrating a network meta-analysis of diabetes trials of sodium glucose cotransporter 2 inhibitors, glucagon-like peptide-1 receptor analogues and dipeptidyl peptidase-4 inhibitors to a representative routine population: a systematic review protocol
Source: BMJ Open. 2022 Oct 27;12(10):e066491. doi: 10.1136/bmjopen-2022-066491 (PMC9621152; doi:10.1136/bmjopen-2022-066491)
Supplement: Author's manuscript [file bmjopen-2022-066491.draft_revisions.pdf]

BMJ Open

BMJ Open is committed to open peer review. As part of this commitment we make the peer review history of every article we publish publicly available.

When an article is published we post the peer reviewers' comments and the authors' responses online. We also post the versions of the paper that were used during peer review. These are the versions that the peer review comments apply to.

The versions of the paper that follow are the versions that were submitted during the peer review process. They are not the versions of record or the final published versions. They should not be cited or distributed as the published version of this manuscript.

BMJ Open is an open access journal and the full, final, typeset and author-corrected version of record of the manuscript is available on our site with no access controls, subscription charges or pay-per-view fees (http://bmjopen.bmj.com).

If you have any questions on BMJ Open's open peer review process please email
info.bmjopen@bmj.com

BMJ Open

Calibrating a network meta-analysis of diabetes trials of sodium glucose co-transporter 2 inhibitors, glucagon-like peptide-1 receptor analogues and dipeptidyl peptidase-4 inhibitors to a representative routine population: a systematic review protocol

Journal:	*BMJ Open*
Manuscript ID	bmjopen-2022-066491
Article Type:	Protocol
Date Submitted by the Author:	07-Jul-2022

Complete List of Authors:	Butterly, Elaine; University of Glasgow Institute of Health and Wellbeing,<br>Wei, Lili; University of Glasgow Institute of Health and Wellbeing<br>Adler, Amanda ; University of Oxford,<br>Almazam, Saleh; University of Glasgow Institute of Health and Wellbeing<br>Alsallumi, Khalid; University of Glasgow Institute of Health and Wellbeing<br>Blackbourn, Luke; University of Edinburgh<br>Dias, Sofia; University of York, Centre for Reviews and Dissemination<br>Hanlon, Peter; University of Glasgow Institute of Health and Wellbeing<br>Hughes, Katherine; NHS Greater Glasgow and Clyde, Department of Diabetes, Glasgow Royal Infirmary<br>Lewsey, Jim; University of Glasgow Institute of Health and Wellbeing<br>Lindsay, Robert; University of Glasgow BHF Glasgow Cardiovascular Research Centre<br>McGurnaghan, Stuart; University of Edinburgh, MRC Institute of Genetics and Cancer<br>Petrie, John; University of Glasgow BHF Glasgow Cardiovascular Research Centre<br>Phillippo, David; University of Bristol<br>Sattar, Naveed; University of Glasgow, Institute of Cardiovascular and Medical Sciences<br>Tomlinson, Laurie; London School of Hygiene and Tropical Medicine, Epidemiology and Population Health<br>Welton, Nicky; University of Bristol<br>Wild, Sarah; University of Edinburgh, Public Health Sciences<br>McAllister, David; University of Glasgow Institute of Health and Wellbeing

Keywords:	Clinical trials < THERAPEUTICS, DIABETES & ENDOCRINOLOGY, EPIDEMIOLOGY, PUBLIC HEALTH

SCHOLARONE™
Manuscripts

Title

Calibrating a network meta-analysis of diabetes trials of sodium glucose co-transporter 2 inhibitors, glucagon-like peptide-1 receptor analogues and dipeptidyl peptidase-4 inhibitors to a representative routine population: a systematic review protocol

Authors

Butterly EW[1], Wei L[1], Adler AI[2], Almazam SA[1], Alsallumi K[1], Blackbourn L[3], Dias S[4], Hanlon P[1], Hughes KA[5], Lewsey J[1], Lindsay RS[1], McGurnaghan S[3], Petrie JR[1], Phillippo DM[6], Sattar N[1], Tomlinson L[7], Welton NJ[6], Wild SH[3] and McAllister DA[1].

Affiliations

1 University of Glasgow, UK

2 Diabetes Trials Unit, University of Oxford, UK

3 University of Edinburgh, UK

4 Centre for Reviews and Dissemination, University of York, UK

5 Dept of Diabetes, Glasgow Royal Infirmary, UK

6 Population Health Sciences, Bristol Medical School, University of Bristol, UK

7 London School of Hygiene and Tropical Medicine, UK

Corresponding author

Professor David A McAllister

Wellcome Trust Intermediate Clinical Fellow, Beit Fellow, Professor of Clinical Epidemiology and Medical Informatics and Consultant in Public Health Medicine

Institute of Health and Wellbeing, University of Glasgow

1 Lilybank Gardens,

Glasgow G12 8RZ

Email: david.mcallister@glasgow.ac.uk

For peer review only - http://bmjopen.bmj.com/site/about/guidelines.xhtml

Abstract (300 words)

Introduction

Participants in randomised controlled trials (trials) are generally younger and healthier than many individuals encountered in clinical practice. Consequently, the applicability of trial findings is often uncertain. To address this, results from trials can be calibrated to more representative data sources. In a network meta-analysis, using a novel approach which allows the inclusion of trials whether or not individual-level participant data (IPD) is available, we will calibrate trials for three drug classes (sodium glucose co-transporter 2 (SGLT2) inhibitors, glucagon-like peptide-1 (GLP1) receptor analogues and dipeptidyl peptidase-4 (DPP4) inhibitors) to the Scottish diabetes register.

Methods and analysis

Medline and EMBASE databases, the US clinical trials registry (clinicaltrials.gov) and the Chinese Clinical Trial Registry (chictr.org.cn) will be searched from 1st January 2002. Two independent reviewers will apply eligibility criteria to identify trials for inclusion. Included trials will be phase 3 or 4 trials of SGLT2 inhibitors, GLP1 receptor analogues or DPP4 inhibitors, with placebo or active comparators, in participants with type 2 diabetes, with at least one of glycaemic control, change in body weight or major adverse cardiovascular event as outcomes. Unregistered trials will be excluded.

We have identified a target population from the population-based Scottish diabetes register. The chosen cohort comprises people in Scotland with type 2 diabetes who either 1) require further treatment due to poor glycaemic control where any of the three drug classes may be suitable, or 2) who have adequate glycaemic control but are already on one of the three drug classes of interest or insulin.

Ethics and dissemination

Ethical approval for IPD use was obtained from the University of Glasgow MVLS College Ethics Committee (Project: 200160070). The Scottish diabetes register has approval from the Scottish A Research Ethics Committee (11/AL/0225) and operates with Public Benefit and Privacy Panel for Health and Social Care approval (1617-0147).

Registration

This systematic review and meta-analysis is registered on PROSPERO (CRD42020184174)

For peer review only - http://bmjopen.bmj.com/site/about/guidelines.xhtml

Strengths and limitations of this study

- This study will estimate the comparative efficacy of SGLT2 inhibitors, GLP1 receptor analogues and DPP4 inhibitors in type 2 diabetes in routine populations using trial calibration, a method which retains the strength of trial data (not breaking randomisation) while improving representativeness using routine healthcare data.
- Unlike other approaches to calibration the planned approach allows more studies to be included in the analysis due to the inclusion of both IPD and aggregate-level trials potentially reducing bias.
- Calibration modelling requires important assumptions, albeit fewer assumptions than simple extrapolation of trial results to wider populations.

Keywords

Clinical trials, generalisability, representativeness, calibration, diabetes

Word count (excluding abstract)

4411

For peer review only

For peer review only - http://bmjopen.bmj.com/site/about/guidelines.xhtml

Introduction

Randomised controlled trials (hereafter abbreviated to trials) are the gold standard for obtaining unbiased estimates of treatment effects. However, trials are limited in terms of representativeness. Trial participants are on average younger, fitter and have fewer comorbid diseases than patients in routine care identified through disease registers, who are the target population for an intervention.[1–5]

In type 2 diabetes, one of the commonest chronic diseases,[6] this gap in representativeness is particularly evident. For example, compared to the population-based Scottish diabetes register, trial participants are on average younger and women are under-represented.[1] This under-representation remains true for trials of the newer glucose-lowering agents (sodium glucose co-transporter 2 (SGLT2) inhibitors, glucagon-like peptide-1 (GLP1) receptor analogues and dipeptidyl peptidase-4 (DPP4) inhibitors). [1,7–11] This problem of under-representation is acknowledged in clinical guidelines, for example the most recent diabetes guidance from the National Institute for Health and Care Excellence (NICE) states “much of the evidence base used to inform this guideline has been generated from studies involving younger adults (study mean ages ranged from 45 to 68 years)”.[6]

However, the clinical implications of this under-representation are not self-evident. Since diabetes complications are commoner in older age groups,[12–14] people in routine care settings may benefit more from treatments than trial participants. Alternatively, since the risk of non-cardiovascular non-diabetes related deaths also increase with age, acting as a competing risk, routine care patients may benefit less than trial participants.[14] Therefore, in routine care settings, the applicability of trial results is uncertain.

Calibrating trial results to make them more representative of target populations in clinical practice is a promising approach to help address this uncertainty. First described by Cole and Stuart,[2] calibration involves re-analysing trial data using the prevalence of baseline characteristics in trial and target populations. Briefly, participants who, compared to the target population, are overrepresented in the trial (e.g. younger people) contribute less to the calibrated treatment effect estimate, while participants who are underrepresented (e.g. older people) contribute more. At the expense of wider confidence intervals for calibrated effects, this “moves” trials in the direction of increased representativeness. Most approaches to calibration respect randomisation and so avoid the confounding by indication which can occur when estimating treatment effects using observational data which is representative of the target population. Calibration also involves fewer assumptions than simpler approaches to extrapolating trial results to target populations.

For peer review only - http://bmjopen.bmj.com/site/about/guidelines.xhtml

Despite these advantages, calibration has not been widely used. Until recently, calibration required individual-level participant data (IPD) (or stratification of results for all levels and every combination of baseline characteristics) for all relevant trials, making it unfeasible in most settings. We propose to overcome this problem by using a novel calibration methodology which incorporates trials where IPD are available and trials where only published summary data are available in a single model. We will use this method to compare SGLT2 inhibitors, GLP1 receptor analogues and DPP4 inhibitors in type 2 diabetes.

Aim

To compare the efficacy of SGLT2 inhibitors, GLP1 receptor analogues and DPP4 inhibitors on glycated haemoglobin (HbA1c), body weight and cardiovascular outcomes in people with established type 2 diabetes by applying a network meta-analysis (NMA) of all relevant type 2 diabetes trials and calibrating to a selected target population from the Scottish diabetes register.

Objectives

1. To compare the efficacy of each drug class as an add on to metformin (dual therapy)
2. To compare the efficacy of each drug class as an add on to metformin plus one other glucose-lowering drug (triple therapy)
3. To compare the efficacy of each drug class singly (monotherapy)

As well as the NMA calibrated to the routine care population, to quantify the impact of the calibration on the final results we will also repeat 1-3 using an uncalibrated NMA and a NMA calibrated to the average population of the trials.

Methods

We plan to conduct a systematic review and calibrated NMA combining results from a model fitted to randomised controlled trials of the relevant drug classes (both IPD and aggregate-level data) with data for a target population defined using the Scottish diabetes register (Figure 1). Here we describe the planned systematic review, planned modelling and the characterisation of the target population.

Systematic review

Eligibility criteria

This review will be performed in keeping with the Preferred Reporting Items for Systematic Reviews and Meta-Analyses statement (PRISMA) statement[15]. We used the PRISMA-P checklist for this protocol.[16] This review has been registered on PROSPERO (CRD42020184174).

For peer review only - http://bmjopen.bmj.com/site/about/guidelines.xhtml

Population

Eligible trials will study people over 18 years old with established type 2 diabetes, with trials of healthy volunteers, people with pre-diabetes or trials that include any other forms of diabetes e.g. type 1 diabetes or gestational diabetes, excluded. There will be no limit placed on duration or severity of diabetes and trials will be included if they examine a sub-population of people with type 2 diabetes defined by a comorbid condition e.g. a trial in people with type 2 diabetes and comorbid fatty liver disease.

Interventions

Eligible trials will study any SGLT2 inhibitor, GLP1 receptor analogue or DPP4 inhibitor as the intervention drug. This will not be limited to drugs approved by regulatory authorities in any specific country. Intervention drug preparations can be short acting or modified release and can be prescribed as mono-, dual- or triple therapy with other glucose lowering drugs including the other two classes of interest plus metformin, sulfonylureas, thiazolidinediones and insulin. Trials will be excluded when the intervention drug was given as a single dose only e.g. peri-operative trials of GLP1 receptor analogues. Trials will be excluded if they were performed under fasting conditions e.g. Ramadan specific trials, as this is likely to have influence on the treatment effect.

Comparators

Trials will be included where the intervention drug was compared to placebo or to an active pharmacological comparator e.g. metformin, sulfonylureas, thiazolidinediones, alpha-glucosidase inhibitors, SGLT2 inhibitors, GLP1 receptor analogues, DPP4 inhibitors, or insulins. Trials will be excluded if they had surgical comparators (e.g. bariatric surgery) or were compared to specific non-pharmacological lifestyle interventions (e.g. very low calorie diets).

Outcomes

The trial outcomes to be analysed are:

1. Glycaemic control- measured by change in glycated haemoglobin (HbA1c) in either % or mmol/mol
2. Change in body weight- measured by weight in kilograms or change in body mass index (BMI)
3. Cardiovascular outcome- measured as composite outcome such as Major Adverse Cardiovascular Events (MACE)

Trials will be included if at least one of the above outcomes was measured. It is not required to be the primary outcome of the trial. Non- inferiority trials will be included.

For peer review only - http://bmjopen.bmj.com/site/about/guidelines.xhtml

Study design

Eligible trials will be limited to randomised phase 3 or 4 trials. Trial registration is required as a marker of trial quality, but no specific registration platform has to be used. There will be no limitation based on trial blinding or enrolment size. Trials of any follow up length will be included except in cases where only HbA1c is reported, in which case these trials will only be included if the follow up length is ≥12 weeks from randomisation reflecting the physiological turnover of red blood cells. Exploratory sub-studies within a trial population e.g. where a small proportion of participants had an additional alternative intervention or exploratory outcome analysed, will be excluded. Other study designs including non-randomised and observational, along with existing meta-analyses, will be excluded.

Information sources

Relevant trials will be identified by systematic searches of Medline and EMBASE (via OVID) databases using a combination of Medical Subject Headings (MeSH) [17]and keyword searches. Terms will be piloted and refined then adapted to each database. (See supplementary appendix for full search strategy)

All searches will be limited from 1st January 2002, as pilot work showed the first phase 3 trials of relevant newer glucose-lowering drugs were all commenced after this date.

To reduce the risk of publication bias, two clinical trials registries (the US clinical trials registry at https://clinicaltrials.gov/[18] and the Chinese Clinical Trial Registry at https://www.chictr.org.cn/[19]) will also be searched for eligible trials using the same criteria.

Data management

The initial review stages will utilise Covidence online software [20] to manage the search records and the screening process. Eligible papers will be saved locally in pdf format and linked to the relevant trial via the corresponding trial registration identifier e.g. nctid. Data extracted from publicly available documents will be processed locally. Where IPD is available this will be processed on Vivli Center for Global Clinical Research Data[21] or Yale University Open Data Access Project (YODA)[22] secure platforms and only approved aggregate level results will be exported and stored on csv files locally. At the time of publication, aggregate level data from the target population and trials will be made available along with sufficient metadata for analysis.

Selection process

Titles and abstracts obtained from the search strategies will be screened by one reviewer for potential relevance. Where the paper is potentially relevant (or if there is uncertainty), a full text

For peer review only - http://bmjopen.bmj.com/site/about/guidelines.xhtml

paper will be acquired and reviewed in the next stage. A random sample of 100 titles and abstracts will be reviewed by an independent reviewer as a quality check. Two reviewers will then both independently review all full text papers and apply eligibility criteria. Reasons for excluding papers will be documented. Where there are conflicts, papers will be discussed in a meeting with at least two reviewers and a joint decision will be documented. Papers will be included if they can be linked to a registered clinical trial meeting the eligibility criteria. The US or Chinese clinical trial registries will also be searched for relevant trials. The online registries will be searched by filtering trial condition as type 2 diabetes mellitus and trial product as any of the three classes of interest. The resulting trials will be screened for relevance using the same criteria used in the database searches. Included trials from the Medline and EMBASE searches, together with additional trials identified on the clinical trial registries, will be collated, and thereafter be identified by a unique trial registration identifier (e.g. a national clinical trial id (nctid) from clinicaltrials.gov).

Data collection process

Descriptive information for each trial (e.g. intervention class, follow up, enrolment) will be extracted from publicly available sources including online trial registries, published papers and study documents. Extraction of trial results will depend on the level of data accessible. The planned calibration analysis incorporates IPD and published trial-level aggregate data, depending on data availability. Two IPD repositories (Vivli and YODA) will be searched for data availability. Where IPD are not available, trial level aggregate data will be collected from publicly available sources. Where the trial is registered to the US Clinical Trial registry at ClinicalTrials.gov, this can be done semi-automatically by interrogation of the Aggregate Analysis of ClinicalTrials.gov (AACT) database[23]. Results extracted from AACT, and any code used, will be checked for accuracy by a second researcher. Where trial results are not available on AACT, they will be double extracted from published documents manually by two researchers.

Data items

The data items to be extracted for each eligible trial are listed in Table 1. This will be individual level for the IPD trials and aggregate level data for the non-IPD trials. In trials where there is crossover or longer term follow up with escape treatment, data will be extracted for the initial randomised period only to reduce confounding by introduction of other agents. Baseline characteristics and outcomes will also be extracted for subgroup populations in trials reporting MACE where these are available.

Effect measures

For the IPD trials, participant level data will be re-analysed, and effect measures calculated. Outcome data from the intention to treat datasets will be extracted where available. For non-IPD

For peer review only - http://bmjopen.bmj.com/site/about/guidelines.xhtml

trials, all available published measures will be extracted including arm level data (e.g. HbA1c at baseline and endpoint or change in HbA1c in each arm) and treatment effect estimates (e.g. ANCOVA). For cardiovascular outcome trials, count data or proportions for each arm will be extracted for the MACE outcome.

Risk of bias

Each eligible trial will be assessed for risk of bias using the Revised Cochrane risk-of-bias tool for randomized trials (RoB 2)[24] using two independent reviewers to assess risk of bias and provide detailed information on confidence of results.

Calibrated NMA

We will perform calibrated NMA for the effect of treatment on glycaemic control, weight loss and cardiovascular outcome of glucose-lowering drugs from the three chosen classes. The formal details of the modelling– which is done within a Bayesian framework – are explained in detail in a separate publication [25], and the use of the modelling for this particular application is described in the supplementary appendix. Briefly, the modelling is conducted in two stages. First, a model is fitted to the trial IPD and trial aggregate level data to estimate treatment effects adjusting for differences in effect modifiers in the trial populations. Next, the fitted model is applied to the target population data to estimate treatment efficacy in that population.

For the main analysis, we will include the following covariates: age, duration of diabetes, HbA1c, estimated glomerular filtration rate (eGFR), total cholesterol, high density lipoprotein cholesterol, systolic blood pressure, diastolic blood pressure, BMI, sex, ethnicity, smoking status, history of cardiovascular disease, history of heart failure, metformin use and insulin use. For the routine data target population, we will use multiple imputation in order to account for missingness. We anticipate low missingness in the IPD therefore we will conduct a complete case analysis for the trial data. In sensitivity analyses we will examine the robustness of the findings to alternative covariate choices.

In the model fitting stage, there is a single underlying individual-level model across all studies, which includes terms for treatment effects, prognostic covariate effects, and treatment-covariate interactions (subgroup effects), with intercepts stratified by study terms for treatment effects and subgroup effects (t-c interactions). This is fitted directly to individuals in the IPD studies, and for the aggregate-level trials the individual-level model is integrated over the trial covariate distribution to obtain the aggregate-level model. Such integration requires information on the distribution of the covariates in each trial (i.e. the distribution of age, sex, BMI etc). Summary level information on the marginal covariate distributions is normally available in the table of baseline characteristics of

For peer review only - http://bmjopen.bmj.com/site/about/guidelines.xhtml

For peer review only

published trial manuscripts; and can be extracted as part of the systematic review and, together with estimated covariances from the IPD studies used to obtain the joint distributions of covariates for each trial. [25,26]

A trial with IPD contributes more information about treatment-covariate interactions than a trial with only aggregate-level data. Nonetheless, both IPD and aggregate-level trials contribute information to the estimation of such interactions. More importantly, the modelling approach enforces consistency for trials with both types of data, and assumes that the relative treatment effects are, conditional on the known covariate levels, similar across trials. This assumption is weaker than the standard assumption in NMA that relative treatment effects are *unconditionally* similar across trials.

If we assume that treatment-covariate interactions are equal (or exchangeable) for treatments within drug-classes, we can estimate these treatment-covariate interactions if there are one or more trials with IPD (or multiple aggregate-level trials across a range of covariate values) within each drug class. This assumption is also weaker than the standard assumption in NMA that there are no covariate-treatment interactions (at least for covariates not identically distributed in all trials).

In the second stage of calibration, the model results are applied to the target population data to estimate treatment efficacy in that population. This is achieved by integrating the estimated regression model for covariate-specific treatment effects over the joint covariate distribution in the target population to obtain population specific treatment effects. This can be done for any target population where information is available for the relevant covariates (or even for any hypothetical target population where an investigator is interested in the treatment effects for any given set of covariate levels).

In this calibrated NMA, for the MACE outcome only, we propose to extend the modelling by including subgroup-level effect estimates for those aggregate trials where subgroup-level data are available for the main covariates of interest. This will further improve precision and potentially allow for the equal interactions within classes assumption to be relaxed. The existing model framework allows inclusion of single subgroups (or fully factorial subgroups where effects are presented for all combinations of the covariates of interest). We will extend the modelling to the more usual scenario where potentially correlated sub-group effects are presented for multiple subgroups.

Target population

A key step in calibration is defining a specific target population of interest. For this research, the Scottish diabetes register has been used to identify the target population. The Scottish diabetes

For peer review only - http://bmjopen.bmj.com/site/about/guidelines.xhtml

register (SCI-diabetes) includes data from >99% of people in Scotland with a diagnosis of diabetes. The data in the Scottish diabetes register platform are extracted regularly from a national database that collates information relevant to diabetes from primary and secondary care that is linked to other datasets via deterministic linkage. Linked datasets include hospitalisations and deaths, prescribing and dispensing data and national renal and cancer registries providing a rich and representative dataset. For this calibration, the 2019 extract from SCI Diabetes was used. The target population comprises people in Scotland with type 2 diabetes who either 1) require further treatment due to inadequate glycaemic control (HbA1c ≥53mmol/mol) where any of the three drug classes may be suitable (i.e. no contraindications of any of the three classes and no alternative focus of treatment e.g. end of life), or 2) have adequate glycaemic control but are already on one of the three drug classes of interest or insulin. People with type 2 diabetes in Scotland alive on 1st January 2019 with diabetes duration of at least one year were first identified (n= 256,620)**.** The cohort was then refined by applying eligibility criteria in a step wise fashion (Section 2, Supplementary Appendix). The criteria were agreed with the steering committee and will be finalised prior to any extraction of results from the clinical trial data. For some of the criteria, for example for body mass index and renal function, several cut-off options were examined to assess their impact on the final population characteristics, and the final decision chosen based on clinical judgement. For the clinical contraindications, diseases were identified via a combination of International Classification of Diseases (ICD) codes[27] from hospital admission data, prescribing data, and outpatient clinic attendance data. Additional details are provided in the supplementary appendix.

Subsequently, we will calibrate the trial results to specific sub-populations within the overall target population to estimate sub-population treatment effects. We will identify these sub-populations using clustering methods alongside clinical judgement and public engagement.

We described the impact of each step in the selection process on the population characteristics using means and standard deviations, medians and interquartile ranges and counts and percentages for symmetrically distributed continuous variables, skewed continuous variables and binary variables respectively.

Patient and public involvement

We have involved a patient and public involvement (PPI) group in the design and funding application stages of this work. We will continue to involve a local PPI group to help guide the subpopulations for calibration and to ensure any outputs are distributed in the best way to help people with diabetes. We have also invited people with diabetes to join our steering committee.

For peer review only - http://bmjopen.bmj.com/site/about/guidelines.xhtml

Results

Target population

We present the details of the target population chosen for calibration to ensure this is documented prior to any data analysis from the clinical trials. Initially, the whole population of people with type 2 diabetes in Scotland alive as of 1st January 2019 with a duration of diabetes of at least one year was identified (n=256,620). This cohort was 56.2% male with a mean age of 66.7 (standard deviation (SD), 12.7) years and had been diagnosed with type 2 diabetes for a mean (SD) of 10.0 (7.1) years. Mean (SD) BMI was 31.7 (6.6) kg/m^2, 14.5% were current smokers and 21.2% had a previous history of cardiovascular disease.

This whole population was then restricted to those defined as eligible for treatment escalation and in whom any of the three classes would be considered, to define the final target population for calibration (n= 127,992). This group was 60.6% male with a mean age of 63.8 (12.1) years and had been diagnosed with type 2 diabetes for a mean of 10.3 (6.9) years. Mean (SD) BMI was 32.7 (6.2) kg/m^2, 14.6% were current smokers and 17.6% had a previous history of cardiovascular disease. (Table 2)

Discussion

This study will use all the available IPD and aggregate-level trial data and data from a diabetes register to estimate the effectiveness of SGLT2 inhibitor, GLP1 receptor analogue and DPP4 inhibitors for patients in routine care settings. It will also be the first study, to our knowledge, to calibrate multiple trials (both IPD and aggregate level) to a representative target population defined using routinely collected healthcare data.

We have opted to perform calibrated NMA to address the differences in characteristics of people with type 2 diabetes in UK clinical practice and participants in trials for the included drug-classes.[1,7–11] A previous study, on applying trial eligibility for a major cardiovascular outcome trial of an SGLT2 inhibitor to a primary care database (n= 1,238,909), found that only 15.7% of people with type 2 diabetes had similar levels of cardiovascular risk to participants in the trial, and only 11.1% of SGLT2 inhibitor treated patients were comparable to the trial participants in terms of baseline characteristics.[9] Similarly, a European study (n= 803, 836) of German, Norwegian, Swedish and Dutch populations found that the proportion who were eligible for the four main SGLT2 inhibitor cardiovascular outcome trials ranged from 17-59%.[10] Similarly, in the USA, a cross-sectional study evaluating the eligibility of people within the Diabetes Collaborative Registry (n=172,643) for SGLT2 inhibitor cardiovascular outcome trials reported that 48% of their population were ineligible for any

For peer review only - http://bmjopen.bmj.com/site/about/guidelines.xhtml

one of the trials, with individual trial eligibility ranging from 26-44%. [8] However, while such studies quantified the degree of lack of representativeness, they do not allow us to assess the likely impact of such differences to the underrepresented populations.

An existing approach to address lack of representativeness is to estimate treatment effects using observational data. Known as pharmaco-epidemiology, such analyses commonly use routine healthcare data, where included individuals are more representative by definition. However, while such studies have mostly yielded results similar to those from comparable clinical trials,[28] they have led to inaccurate conclusions in several cases; this is because pharmaco-epidemiological analyses are limited by the problem of confounding by indication; treated and untreated patients differ in their susceptibility to disease-related outcomes and it is currently not possible to determine when such analyses have successfully overcome confounding by indication.[29–32]

Another existing approach commonly used in health technology assessments is to use simple extrapolation, wherein relative treatment effects from clinical trials are combined with data on event rates from other (ideally representative) data sources to estimate benefits and harms.[33] For example, on applying a relative risk of death of 0.80 from a clinical trial to a target population with one-year mortality of 10%, the expected absolute risk reduction for that target population is 2%. A strength of using simple extrapolation is that doing so avoids the problem of confounding by indication. However, in this approach one must assume that treatment effects are similar in trial and routine care populations regardless of differences in patient characteristics. This assumption can rarely be justified on biological or clinical grounds.

Trial calibration has the advantage over pharmaco-epidemiology that it avoids confounding by indication (as it does not break randomisation). However, it also has an advantage over simple extrapolation that it does not require the assumption that treatment effects are similar in trial and routine care settings, but only that treatment effects are similar for participants who are similar with respect to characteristics included in the modelling (e.g. with similar age, sex, BMI, etc). This assumption allows greater confidence in applying trial data to routine care settings suggesting that, alongside other methodologies (including pharmaco-epidemiology) it may have a valuable role in assessing the likely applicability of trial findings to participants in routine care settings.

The particular form of trial calibration we propose to use – calibrated NMA via multilevel network meta-regression– was recently developed to address limitations of conventional NMA (combining IPD and aggregate-level data in a coherent manner, whilst exploring and explaining heterogeneity in treatment effects according to differences in participant characteristics within and across trials) by co-authors (DP, NW, SD). We are not aware that this approach has previously been used to address

For peer review only - http://bmjopen.bmj.com/site/about/guidelines.xhtml

the under-representation within clinical trials of participants with certain characteristics. This approach to calibrated NMA has a number of advantages. First, it allows us to calibrate trial findings to any target population, including sub-populations of clinical interest, provided one is willing to assume that treatment effects are similar for participants who are similar with respect to characteristics included in the modelling. Indeed, it even allows us to calibrate treatment effects to notional populations, allowing us to explore the sensitivity of trial findings under any plausible set of patient characteristics. Perhaps more importantly, the method allows us to use all relevant trials, whether or not IPD are available. This is likely to result in greater precision and less bias than methods which require access to individual-level participant data for all calibrated trials.

Limitations

Calibrated NMA is a potentially useful approach for assessing the applicability of trial findings to target populations in routine care settings, however, in addition to the assumptions stated above there are a number of limitations. First, if target populations and trial participants differ, even after conditioning on known characteristics, with respect to other characteristics which modify treatment effects, the calibrated treatment effects may be biased. Secondly, even where all important characteristics are included in the modelling, if they are incorrectly parameterised this may also cause misleading findings (e.g. if the true treatment-covariate interaction is non-linear and it is modelled with a linear term). Finally, some participants are entirely excluded from clinical trials, not simply under-represented (e.g. those with very severe frailty, extensive multimorbidity, or overwhelming personal circumstances such as severe dependence syndromes). While it may be technically possible to calibrate trial results to these groups (e.g. by extrapolation), findings from such analyses are likely to be misleading.

Conclusion

This systematic review and calibrated NMA will assess the efficacy of newer glucose lowering agents in type 2 diabetes, producing results more generalisable to routine care populations. This has the potential to influence clinical practice as well as to provide an exemplar of the value of trial calibration methods.

Ethics and dissemination

Ethical approval for use of individual participant level data was obtained from the University of Glasgow MVLS College Ethics Committee (Project No: 200160070). The Scottish diabetes register has approval from the Scottish A Research Ethics Committee (ref 11/AL/0225) and operates with Public Benefit and Privacy Panel for Health and Social Care approval (ref 1617-0147).

For peer review only - http://bmjopen.bmj.com/site/about/guidelines.xhtml

Author contributions

DM, DP, SD and NW designed the study and EB and LW designed the search strategy. EB, LW and DK will conduct the systematic review screening. EB, LW, KA and SA will conduct the risk of bias scoring. EB conducted the analysis within the Scottish diabetes register, supervised by DM. EB and DM wrote the first draft of this protocol paper. All authors reviewed, commented on, and made changes on the final protocol and manuscript.

Funding

This research is funded by the Medical Research Council (MR/T017112/1: Routine care treatment effectiveness in people with type 2 diabetes: Maximising the applicability of clinical trials)

David McAllister is funded via an Intermediate Clinical Fellowship and Beit Fellowship from the Wellcome Trust.

None of the funders had any influence over the study design, analysis, or decision to submit for publication.

Acknowledgements

We would like to acknowledge the people with diabetes in Scotland whose data make the diabetes register and the healthcare professionals who record these data while providing healthcare.

We would also like to thank the University of Glasgow Medical and Veterinary Life Sciences patient and public involvement and engagement group for their input regarding this study.

Conflict of interests

There are no conflicts of interest to declare.

References

1. Saunders C, Byrne CD, Guthrie B, Lindsay RS, McKnight JA, Philip S, et al. External validity of randomized controlled trials of glycaemic control and vascular disease: how representative are participants? Diabet Med J Br Diabet Assoc. 2013 Mar;30(3):300–8.

2. Cole SR, Stuart EA. Generalizing evidence from randomized clinical trials to target populations: The ACTG 320 trial. Am J Epidemiol. 2010 Jul 1;172(1):107–15.

3. Coca SG, Krumholz HM, Garg AX, Parikh CR. Underrepresentation of renal disease in randomized controlled trials of cardiovascular disease. JAMA. 2006 Sep 20;296(11):1377–84.

4. Brown N, Melville M, Gray D, Young T, Skene AM, Wilcox RG, et al. Relevance of clinical trial results in myocardial infarction to medical practice: comparison of four year outcome in

For peer review only - http://bmjopen.bmj.com/site/about/guidelines.xhtml

For peer review only

participants of a thrombolytic trial, patients receiving routine thrombolysis, and those deemed ineligible for thrombolysis. Heart Br Card Soc. 1999 Jun;81(6):598–602.

5. Udell JA, Wang TY, Li S, Kohli P, Roe MT, de Lemos JA, et al. Clinical Trial Participation After Myocardial Infarction in a National Cardiovascular Data Registry. JAMA. 2014 Aug 27;312(8):841–3.

6. Type 2 diabetes in adults: management | Guidance and guidelines | NICE [Internet]. [cited 2019 Feb 22]. Available from: https://www.nice.org.uk/guidance/ng28

7. Pintat S, Fenici P, Hammar N, Ji L, Khunti K, Medina J, et al. Eligibility of patients with type 2 diabetes for sodium–glucose cotransporter 2 inhibitor cardiovascular outcomes trials: a global perspective from the DISCOVER study. BMJ Open Diabetes Res Care. 2019 Mar 1;7(1):e000627.

8. Wittbrodt E, Chamberlain D, Arnold SV, Tang F, Kosiborod M. Eligibility of patients with type 2 diabetes for sodium-glucose co-transporter-2 inhibitor cardiovascular outcomes trials: An assessment using the Diabetes Collaborative Registry. Diabetes Obes Metab. 2019;21(8):1985–9.

9. McGovern A, Feher M, Munro N, de Lusignan S. Sodium-Glucose Co-transporter 2 (SGLT2) Inhibitor: Comparing Trial Data and Real-World Use. Diabetes Ther Res Treat Educ Diabetes Relat Disord. 2017 Apr;8(2):365–76.

10. Birkeland KI, Jørgensen ME, Carstensen B, Persson F, Gulseth HL, Thuresson M, et al. Cardiovascular mortality and morbidity in patients with type 2 diabetes following initiation of sodium-glucose co-transporter-2 inhibitors versus other glucose-lowering drugs (CVD-REAL Nordic): a multinational observational analysis. Lancet Diabetes Endocrinol. 2017 Sep 1;5(9):709–17.

11. Weng. A Distribution-based Method for Assessing The Differences between Clinical Trial Target Populations and Patient Populations in Electronic Health Records. Appl Clin Inform. 2014;5:463–479.

12. Read SH, McAllister DA, Colhoun HM, Farran B, Fischbacher C, Kerssens JJ, et al. Incident ischaemic stroke and Type 2 diabetes: trends in incidence and case fatality in Scotland 2004-2013. Diabet Med J Br Diabet Assoc. 2018;35(1):99–106.

13. Read SH, Kerssens JJ, McAllister DA, Colhoun HM, Fischbacher CM, Lindsay RS, et al. Trends in type 2 diabetes incidence and mortality in Scotland between 2004 and 2013. Diabetologia. 2016;59(10):2106–13.

14. Tancredi M, Rosengren A, Svensson AM, Kosiborod M, Pivodic A, Gudbjörnsdottir S, et al. Excess Mortality among Persons with Type 2 Diabetes. N Engl J Med. 2015 Oct 29;373(18):1720–32.

15. The PRISMA 2020 statement: an updated guideline for reporting systematic reviews | The BMJ [Internet]. [cited 2022 May 16]. Available from: https://www.bmj.com/content/372/bmj.n71

16. Moher D, Shamseer L, Clarke M, Ghersi D, Liberati A, Petticrew M, et al. Preferred reporting items for systematic review and meta-analysis protocols (PRISMA-P) 2015 statement. Syst Rev. 2015 Jan 1;4(1):1.

For peer review only

For peer review only - http://bmjopen.bmj.com/site/about/guidelines.xhtml

17. Home - MeSH - NCBI [Internet]. [cited 2022 May 16]. Available from: https://www.ncbi.nlm.nih.gov/mesh/

18. Home - ClinicalTrials.gov [Internet]. [cited 2020 Jul 1]. Available from: https://clinicaltrials.gov/

19. Chinese Clinical Trial Register (ChiCTR) - The world health organization international clinical trials registered organization registered platform [Internet]. [cited 2020 Jul 1]. Available from: http://www.chictr.org.cn/abouten.aspx

20. Covidence - Better systematic review management [Internet]. Covidence. [cited 2022 May 16]. Available from: https://www.covidence.org/

21. Vivli - Center for Global Clinical Research Data [Internet]. [cited 2022 May 16]. Available from: https://vivli.org/

22. The YODA Project [Internet]. [cited 2022 May 16]. Available from: https://yoda.yale.edu/

23. AACT Database | Clinical Trials Transformation Initiative [Internet]. [cited 2022 May 16]. Available from: https://aact.ctti-clinicaltrials.org/

24. RoB 2: A revised Cochrane risk-of-bias tool for randomized trials | Cochrane Bias [Internet]. [cited 2022 May 16]. Available from: https://methods.cochrane.org/bias/resources/rob-2-revised-cochrane-risk-bias-tool-randomized-trials

25. Phillippo DM, Dias S, Ades AE, Belger M, Brnabic A, Schacht A, et al. Multilevel network meta-regression for population-adjusted treatment comparisons. J R Stat Soc Ser A Stat Soc. 2020;183(3):1189–210.

26. Phillippo DM, Dias S, Ades AE, Welton NJ. Assessing the performance of population adjustment methods for anchored indirect comparisons: A simulation study. Stat Med. 2020;39(30):4885–911.

27. ICD-10 Version:2019 [Internet]. [cited 2022 May 16]. Available from: https://icd.who.int/browse10/2019/en

28. Anglemyer A, Horvath HT, Bero L. Healthcare outcomes assessed with observational study designs compared with those assessed in randomized trials. Cochrane Database Syst Rev. 2014 Apr 29;(4):MR000034.

29. Bosco JLF, Silliman RA, Thwin SS, Geiger AM, Buist DSM, Prout MN, et al. A most stubborn bias: no adjustment method fully resolves confounding by indication in observational studies. J Clin Epidemiol. 2010 Jan;63(1):64–74.

30. Hue TF, Cummings SR, Cauley JA, Bauer DC, Ensrud KE, Barrett-Connor E, et al. Effect of Bisphosphonate Use on Risk of Postmenopausal Breast Cancer. JAMA Intern Med. 2014 Oct;174(10):1550–7.

31. Rossouw JE, Anderson GL, Prentice RL, LaCroix AZ, Kooperberg C, Stefanick ML, et al. Risks and benefits of estrogen plus progestin in healthy postmenopausal women: principal results From the Women's Health Initiative randomized controlled trial. JAMA. 2002 Jul 17;288(3):321–33.

For peer review only

For peer review only - http://bmjopen.bmj.com/site/about/guidelines.xhtml

32. Criner GJ, Connett JE, Aaron SD, Albert RK, Bailey WC, Casaburi R, et al. Simvastatin for the Prevention of Exacerbations in Moderate-to-Severe COPD. N Engl J Med. 2014 Jun 5;370(23):2201–10.

33. Dias S, Welton NJ, Sutton AJ, Ades AE. NICE DSU Technical Support Document 1: Introduction to Evidence Synthesis for Decision Making. National Institute for Health and Clinical Excellence; 2011. (NICE DSU Technical Support Document in Evidence Synthesis).

For peer review only

Table 1: Data variables for extraction

Individual level data/Aggregate level data		Trial descriptive data
**Baseline characteristics/ target population characteristics**	**Trial Outcomes**	**Trial Details**
• Age in years<br>• Sex<br>• Race/Ethnicity<br>• Geographical location<br>• Duration of type 2 diabetes mellitus in years<br>• Known cardiovascular disease- does the participant have history of atherosclerotic cardiovascular disease (coronary, cerebrovascular or peripheral vascular disease- see ICD10 codes below) (y/n)<br>• Use of any concomitant glucose lowering agents at baseline (y/n)<br>• Use of metformin at baseline (y/n)<br>• Use of sulphonylurea at baseline (y/n)<br>• Use of thiazolidinediones at baseline (y/n)<br>• Use of DPP4 inhibitor at baseline (y/n)<br>• Use of GLP1 receptor agonist at baseline (y/n)<br>• Use of SGLT2 inhibitor at baseline (y/n)<br>• Use of insulin at baseline (y/n)<br>• Smoking status- Current smoker (y/n)<br>• eGFR (document what calculation used if this info is available)<br>• Serum creatinine (in case eGFR needs to be calculated)<br>• Urinary albumin creatinine	• HbA1c (% or mmol/mol)<br>• Body mass index (kg/m2)<br>• Body weight and height (kg, m)<br>• Cardiovascular outcome: MACE composite endpoint where available along with individual components (usually cardiovascular death, non-fatal myocardial infarction, and non-fatal stroke- but check study documents) and hospitalisation for heart failure where available<br>• Adverse events<br><br>**Note**: *All variables will be extracted in available units (which will be recorded) then can subsequently converted onto the desired scale.	• Trial identifier<br>• Trial title<br>• Brief description<br>• Phase<br>• Single vs multicentre trial<br>• Geographical location of trial<br>• Study design (e.g., blinded)<br>• Sponsor (e.g., Industry sponsor)<br>• Date of trial completion<br>Eligibility<br>• Inclusion/exclusion criteria<br>• Study enrolment number<br>Intervention<br>• Intervention drug (generic names)<br>• Comparator drug(s)/regimes<br>• Dosage and frequency of medications<br>• Other drugs allowed in arms<br>• Duration of follow up for each outcome independently

For peer review only

For peer review only - http://bmjopen.bmj.com/site/about/guidelines.xhtml

ratio in mg/g<br>• Total/HDL/LDL Cholesterol in mmol/l<br>• Systolic blood pressure (mmHg)<br>• Diastolic blood pressure (mmHg)<br>• Body weight (kg)<br>• Height (m)	ICD10 codes to identify cardiovascular disease:<br>ICD10: I20.0, I20.1, I20.8, I20.9, I21.0, I21.1, I21.2, I21.3, I21.4, I21.9, I22.0, I22.1, I22.8, I22.9, I23.0, I23.1, I23.2, I23.3, I23.4, I23.5, I23.6, I23.8, I24.0 I24.1, I24.8, I24.9, I25.0, I25.1, I25.2, I25.3, I25.4, I25.5, I25.6, I25.8, I25.9, I63.0, I63.1, I63.2, I63.3, I63.4, I63.5, I63.6, I63.8, I63.9, I64.0, I65.0 , I65.1, I65.2, I65.3, I65.8, I65.9, I66.0, I66.1, I66.2, I66.3, I66.4, I66.8, I66.9, I67.2, I67.8, I67.9, I69.3, I69.4, I69.8, I70.0, I70.1, I70.2, I70.8, I70.9 , I73.0, I73.1, I73.8, I73.9	

Table 1: Data to be collected from routine data and trial data

For peer review only - http://bmjopen.bmj.com/site/about/guidelines.xhtml

Table 2 Summary characteristics of the Scottish target population

For peer review only

	Whole Scottish type 2 diabetes population ≥1 year post diagnosis	Defined Scottish Target Population for Calibration
n	256,620	127,992
n (%) male	144,338 (56.2%)	77,599 (60.6%)
Mean (SD) age in years	66.7 (12.7)	63.8 (12.1)
Mean (SD) body mass index in kg/m^2	31.7 (6.6)	32.7(6.2)
Mean (SD) duration diabetes in years	10.0 (7.1)	10.3 (6.9)
Mean (SD) HbA1c in mmol/mol; %	60.2 (15.1). 7.7% (1.7%)	67.4 (13.1). 8.3% (1.2%)
Mean (SD) estimated glomerular filtration rate in $ml/min/1.73m^2$	77.6 (21.3)	81.7 (19.9)
Mean (SD) total cholesterol in mmol/l	4.3 (1.0)	4.4 (1.0)
Mean (SD) high density lipoprotein cholesterol in mmol/l	1.2 (0.3)	1.1 (0.3)
Mean (SD) systolic blood pressure in mmHg	135.5 (12.2)	135.7 (11.9)
Mean (SD) diastolic blood pressure in mmHg	76.8 (7.6)	77.8 (7.6)
White racial group %	75.8%	75.6%
Asian racial group %	3.8%	4.2%
Black racial group %	0.5%	0.5%
Mixed or other racial group %	3.0%	3.1%
Race unknown %	17.0%	16.5%
% Current smokers	14.5%	14.6%
% History of heart failure	11.9%	9.1%
% History of cardiovascular disease	21.2%	17.6%
% Metformin use	55.2%	69.2%
% Insulin use	8.4%	10.3%

Table 2: Summary characteristics of the whole population within the Scottish diabetes register who have been diagnosed with type 2 diabetes for at least 1 year and a subset who are the defined target population. Missingness of these individual variables in the target population are as follows: sex (0%), age (0%), body mass index (3.4%), duration (0%), glycated

For peer review only - http://bmjopen.bmj.com/site/about/guidelines.xhtml

haemoglobin (HbA1c) (1.2%), estimated glomerular filtration rate (1.5%), total cholesterol (2.1%), high density lipoprotein cholesterol (7.4%), systolic blood pressure (1.1%), diastolic blood pressure (1.1%), smoking status (7.4%)

For peer review only

For peer review only - http://bmjopen.bmj.com/site/about/guidelines.xhtml

Figure 1: Overview of outputs

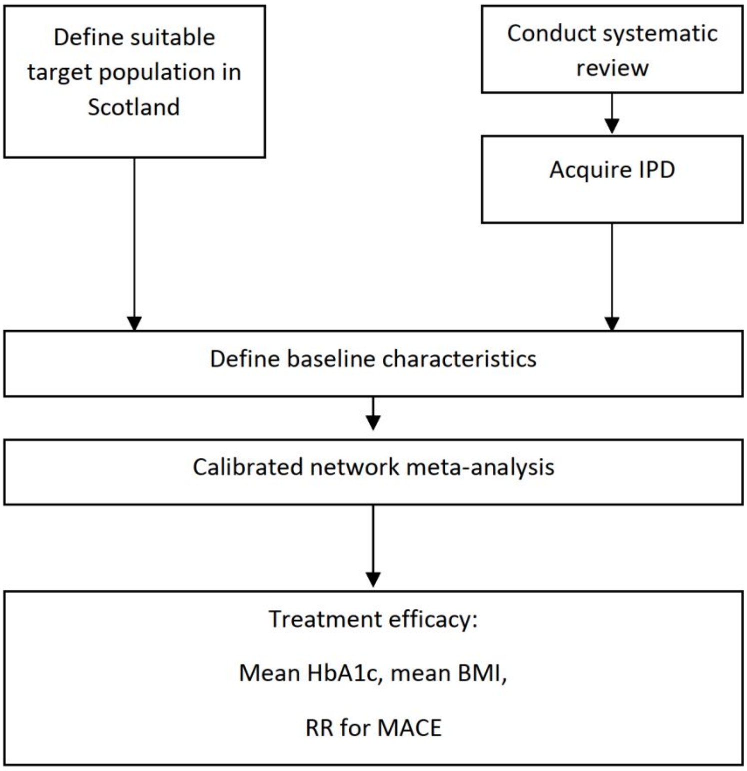

IPD – individual level participant data

HbA1c- glycated haemoglobin

RR- relative risk measures

MACE- composite measure of major adverse cardiovascular event

For peer review only - http://bmjopen.bmj.com/site/about/guidelines.xhtml

Supplementary appendix

Section 1: Search strategies

Medline database

Table S1: Medline search strategy

# ▲	Searches	Results
1	non-insulin dependent diabetes mellitus/	125129
2	glucose intolerance/	8257
3	diabetic obesity/	0
4	impaired glucose tolerance/	8257
5	(non-insulin* depend* or noninsulin* depend* or noninsulindepend* or non insulindepend*).tw.	12075
6	((typ* 2 or typ* II) adj4 diabet*).tw.	132687
7	((adult* or matur* or late or slow or stabl* or obes*) adj4 diabet*).tw.	53350
8	(T2D* or DM2 or IIDM or MODY or NIDDM).tw.	35508
9	((nonketo* or non keto* or ketoresist* or keto resist*) adj4 diabet*).tw.	491
10	impaired glucose toleran*.tw.	10516
11	glucose intoleran*.tw.	10077
12	insulin* resistan*.tw.	76852
13	(insulin* defic* adj2 relativ*).tw.	184
14	(metabolic* syndrom* or plurimetabolic* syndrom*).tw.	46774
15	glucagon like peptide 1 receptor agonist/	0
16	(glucagon-like peptide 1 receptor inhibitor* or glucagon-like peptide 1 receptor agonist* or glucagon-like peptide 1 inhibitor* or glucagon-like peptide 1 agonist* or GLP-1 receptor inhibitor* or GLP-1 receptor agonist* or GLP-1 inhibitor* or GLP-1 agonist*).tw.	3107
17	albiglutide/	0
18	dulaglutide/	0
19	exendin 4/	2290
20	liraglutide/	1479
21	lixisenatide/	0
22	semaglutide/	0
23	taspoglutide/	0

For peer review only - http://bmjopen.bmj.com/site/about/guidelines.xhtml

24	albiglutide.tw.	168
25	dulaglutide.tw.	260
26	(exenatide or exendin 4).tw.	3063
27	liraglutide.tw.	2254
28	lixisenatide.tw.	342
29	semaglutide.tw.	254
30	taspoglutide.tw.	56
31	dipeptidyl peptidase IV inhibitor/	3609
32	(dipeptidyl-peptidase IV Inhibitor* or dipeptidyl-peptidase 4 Inhibitor* or ((DPP4 or DPP 4 or DPP IV) adj inhibitor*)).tw.	4570
33	alogliptin/	0
34	anagliptin/	0
35	gemigliptin/	0
36	linagliptin/	369
37	omarigliptin/	0
38	saxagliptin/	0
39	sitagliptin/	1300
40	teneligliptin/	0
41	vildagliptin/	592
42	alogliptin.tw.	419
43	anagliptin.tw.	59
44	gemigliptin.tw.	49
45	linagliptin.tw.	615
46	omarigliptin.tw.	37
47	saxagliptin.tw.	594
48	sitagliptin.tw.	2029
49	teneligliptin.tw.	116
50	vildagliptin.tw.	904
51	evogliptin.tw.	19
52	evogliptin/	0
53	sodium glucose cotransporter 2 inhibitor/	0
54	(sodium glucose transporter 2 inhibitor* or sodium glucose transporter ii inhibitor* or SGLT 2 inhibitor*).tw.	454
55	(sodium glucose cotransporter adj3 inhibitor*).tw.	1192
56	(sodium glucose co transporter adj3 inhibitor*).tw.	719
57	canagliflozin/	515
58	dapagliflozin/	0
59	empagliflozin/	0
60	ertugliflozin/	0
61	tofogliflozin/	0
62	canagliflozin.tw.	789
63	dapagliflozin.tw.	818
64	empagliflozin.tw.	862

For peer review only - http://bmjopen.bmj.com/site/about/guidelines.xhtml

65	ertugliflozin.tw.	61
66	tofogliflozin.tw.	86
67	ipragliflozin/	0
68	ipragliflozin.tw.	166
69	Randomized Controlled Trials as Topic/	126623
70	randomized controlled trial/	490217
71	Random Allocation/	100528
72	Double Blind Method/	153484
73	Single Blind Method/	27365
74	clinical trial/	518141
75	clinical trial, phase i.pt.	19368
76	clinical trial, phase ii.pt.	31271
77	clinical trial, phase iii.pt.	15567
78	clinical trial, phase iv.pt.	1754
79	controlled clinical trial.pt.	93274
80	randomized controlled trial.pt.	490217
81	multicenter study.pt.	257365
82	clinical trial.pt.	518141
83	exp Clinical Trials as topic/	330616
84	(clinical adj trial$).tw.	334395
85	((singl$ or doubl$ or treb$ or tripl$) adj (blind$3 or mask$3)).tw.	164050
86	PLACEBOS/	34468
87	placebo$.tw.	204278
88	randomly allocated.tw.	26477
89	(allocated adj2 random$).tw.	29629
90	or/69-89	1553327
91	or/1-14	295318
92	or/15-30	6907
93	or/31-52	7002
94	or/53-68	3313
95	92 or 93 or 94	15069
96	90 and 91 and 95	4292
97	case report.tw.	289645
98	letter/	1036792
99	historical article/	354203
100	or/97-99	1665591
101	96 not 100	4237
102	limit 101 to human	3612
103	limit 102 to english language	3452
104	limit 103 to yr="2002-Current"	3452

For peer review only - http://bmjopen.bmj.com/site/about/guidelines.xhtml

Embase database

Table S2: Embase search strategy

▲	Searches	Results
1	((diabetes or diabetes mellitus or diabetic*) adj1 (type 2 or type II or type ii or non-insulin dependent or noninsulin dependent or adult onset or mature onset or late onset)).tw	219042
2	(diabetic nephropath* or diabetic kidney disease).tw.	26837
3	glucose intolerance/	17965
4	diabetic obesity/	3737
5	impaired glucose tolerance/	30222
6	(non insulin* depend* or noninsulin* depend* or noninsulindepend* or non insulindepend*).tw.	14344
7	((typ* 2 or typ* II) adj4 diabet*).tw.	213585
8	((adult* or matur* or late or slow or stabl* or obes*) adj4 diabet*).tw.	85106
9	(T2D* or DM2 or IIDM or MODY or NIDDM).tw.	64908
10	((nonketo* or non keto* or ketoresist* or keto resist*) adj4 diabet*).tw.	721
11	impaired glucose toleran*.tw.	16435
12	glucose intoleran*.tw.	15372
13	insulin* resistan*.tw.	116387
14	(insulin* defic* adj2 relativ*).tw.	310
15	(metabolic* syndrom* or plurimetabolic* syndrom*).tw.	76726
16	glucagon like peptide 1 receptor agonist/	4508
17	(glucagon-like peptide 1 receptor inhibitor* or glucagon-like peptide 1 receptor agonist* or glucagon-like peptide 1 inhibitor* or glucagon-like peptide 1 agonist* or<br>GLP-1 receptor inhibitor* or GLP-1 receptor agonist* or GLP-1 inhibitor* or GLP-1 agonist*).tw.	5749
18	albiglutide/	871
19	dulaglutide/	1104
20	exendin 4/	9969
21	liraglutide/	7911
22	lixisenatide/	1317
23	semaglutide/	861
24	taspoglutide/	254
25	albiglutide.tw.	332
26	dulaglutide.tw.	654
27	(exenatide or exendin 4).tw.	5902
28	liraglutide.tw.	4839
29	lixisenatide.tw.	693
30	semaglutide.tw.	492
31	taspoglutide.tw.	110
32	dipeptidyl peptidase IV inhibitor/	8745

For peer review only - http://bmjopen.bmj.com/site/about/guidelines.xhtml

33	(dipeptidyl-peptidase IV Inhibitor* or dipeptidyl-peptidase 4 Inhibitor* or ((DPP4 or DPP 4 or DPP IV) adj inhibitor*)).tw.	8196
34	alogliptin/	1740
35	anagliptin/	198
36	gemigliptin/	171
37	linagliptin/	2337
38	omarigliptin/	116
39	saxagliptin/	2941
40	sitagliptin/	7976
41	teneligliptin/	338
42	vildagliptin/	3759
43	alogliptin.tw.	739
44	anagliptin.tw.	122
45	gemigliptin.tw.	110
46	linagliptin.tw.	1304
47	omarigliptin.tw.	59
48	saxagliptin.tw.	1226
49	sitagliptin.tw.	4148
50	teneligliptin.tw.	238
51	vildagliptin.tw.	1744
52	evogliptin.tw.	37
53	evogliptin/	48
54	sodium glucose cotransporter 2 inhibitor/	3435
55	(sodium glucose transporter 2 inhibitor* or sodium glucose transporter ii inhibitor* or SGLT 2 inhibitor*).tw.	933
56	(sodium glucose cotransporter adj3 inhibitor*).tw.	1827
57	(sodium glucose co transporter adj3 inhibitor*).tw.	1295
58	canagliflozin/	2568
59	dapagliflozin/	2975
60	empagliflozin/	2877
61	ertugliflozin/	307
62	tofogliflozin/	286
63	canagliflozin.tw.	1536
64	dapagliflozin.tw.	1926
65	empagliflozin.tw.	1803
66	ertugliflozin.tw.	143
67	tofogliflozin.tw.	166
68	ipragliflozin/	484
69	ipragliflozin.tw.	284
70	or/1-15	455561
71	or/16-31	19351
72	or/32-53	19026
73	or/54-69	9181

For peer review only

For peer review only - http://bmjopen.bmj.com/site/about/guidelines.xhtml

74	71 or 72 or 73	38216
75	Clinical Trial/	984789
76	Randomized Controlled Trial/	576557
77	controlled clinical trial/	465986
78	multicenter study/	231443
79	Phase 3 clinical trial/	43062
80	Phase 4 clinical trial/	3641
81	exp RANDOMIZATION/	85032
82	Single Blind Procedure/	36861
83	Double Blind Procedure/	169262
84	Crossover Procedure/	61406
85	PLACEBO/	353947
86	randomi?ed controlled trial$.tw.	213219
87	rct.tw.	34296
88	(random$ adj2 allocat$).tw.	41310
89	single blind$.tw.	23926
90	double blind$.tw.	209043
91	((treble or triple) adj blind$).tw.	1070
92	placebo$.tw.	303630
93	Prospective Study/	557933
94	or/75-93	2246792
95	Case Study/	73901
96	case report.tw.	420554
97	abstract report/ or letter/	1124332
98	Conference proceeding.pt.	0
99	Conference abstract.pt.	3581657
100	Editorial.pt.	633720
101	Letter.pt.	1089718
102	Note.pt.	774711
103	or/95-102	6547596
104	94 not 103	1690232
105	70 and 74 and 104	6157
106	limit 105 to human	6042
107	limit 106 to english language	5816
108	limit 107 to yr="2002 -Current"	5812

For peer review only

For peer review only - http://bmjopen.bmj.com/site/about/guidelines.xhtml

Section 2: Protocol for routine healthcare data target population

Scope:

The scope of this document is to set out a protocol for identifying a clinically appropriate target population for calibration modelling within the routine datasets.

Aim:

1) To identify a clinically appropriate target population within the Scottish diabetes register for calibration modelling of a large network meta-analysis of glucose lowering drugs
2) Document the variables to be collected and summarised within the identified population

Background

For the proposed calibration modelling to be clinically relevant, the routine data target population to which the models are applied requires to be clearly set out and clinically justifiable. Using a 2019 extract of the SCI-diabetes database we aim to identify a population of people with type 2 diabetes mellitus where prescription of any of the three drug classes of interest (Sodium Glucose Co-Transporter 2 Inhibitors (SGLT2i) /Glucagon-Like Peptide 1 Receptor Agonists (GLP1ra) / Dipeptidyl Peptidase-4 Inhibitors (DPP4i)) would realistically be considered should the individual require treatment escalation. We aim to exclude anyone who would be considered to have a significant contraindication to any of the three drug classes.

Subsequent work will include clustering to identify more specific subsets of the population e.g., based on age, sex, body weight, renal function, cardiovascular risk. This will allow calibration to more specific subsets of the overall target population. This will be described in a later document.

Pilot work

We conducted some exploratory searches of the 2017 extract of SCI-diabetes to help guide this protocol. We identified those within the register who were prescribed at least one of the drug classes of interest. Overall, we identified 56,867 people on at least one of the target drugs. (Mean age 64.65 years, weight 98.14kg, glycated haemoglobin (HbA1c) 66.97mmol/mol, systolic blood pressure 136.55mmHg, estimated glomerular filtration rate (eGFR) 58.45. ml/min/1.73m^2).

Proposed steps

1) Access the 2019 data extract- and familiarise with datasets available within and data included in each.
2) Limit included participants to those where absolute contraindications for proposed drug classes are absent (see specific exclusions).
3) Extract data on descriptive variables from Table 1 where available.
4) Continuous observations for each individual will be taken as the mean of measurements over 3 years prior to 1/1/19. The most recent measurement in last 3 years will be taken for categorical variables e.g. smoking.
5) If all of the following variables are missing for the last 3 years, we will presume likely that the individual has either moved away or is not engaged with clinical services and they will not be included: HbA1c, systolic blood pressure, diastolic blood pressure, smoking status, fasting plasma glucose, urinary albumin creatinine ratio, total cholesterol, high density lipoprotein cholesterol, low density lipoprotein cholesterol, eGFR and body mass index.

For peer review only - http://bmjopen.bmj.com/site/about/guidelines.xhtml

6) Previous comorbidities/prescriptions will be extracted as present if appear in previous 10 years of data.
7) Comorbidity data will be defined using ICD10 codes within the linked SMR01 dataset (specified below). As per large cardiovascular outcome trials e.g. CANVAS[1], history of cardiovascular disease will be defined as history of atherosclerotic cardiovascular disease including coronary, cerebrovascular or peripheral vascular disease.
8) Comorbidity data from SMR01/prescribing data will be included where the comorbidity appears in any position in the discharge data e.g., primary diagnosis or any other position of diagnosis
9) Create preliminary definition of overall population to be used for calibration based on above which may include modification of variables collected based on availability.
10) Provide summary statistics including number included/excluded to steering committee and (PRIOR to performing calibration or running NMA model on trial data) amend target population protocol on basis of feedback.

Proposed population defining characteristics

Timeframe:

- Date of diagnosis of type 2 diabetes mellitus at least one year prior to 1st Jan 2019
- Limit comorbidity data to a ten year look back
- Must be alive at time of extraction therefore exclude if death on or before chosen date (1st January 2019)

Age:

- Limit to 18 years old or above on 1st Jan 2019 or at diagnosis of diabetes
- No upper limits based on current age or age at diagnosis

Sex:

- No limits based on sex

Diagnosis:

- Must have documented diagnosis of type 2 diabetes mellitus within the derived diagnosis variable in dataset diagnosed before or on 1/1/18
- There will be no limits to the duration of diabetes diagnosis
- Those with diabetes in remission will be excluded when HbA1c limit applied.

Glycaemic control:

- No limit to HbA1c at diagnosis
- Limit population to those with most recent HbA1c ≥53mmol/mol or those with HbA1c <53mmol/mol but currently on one of the three drug classes of interest, or insulin.

Body Mass Index:

- Limit to those with most recent BMI measurement to ≥23.5kg/m^2 (use cleaned variable either from clinician entered variable from Sci Diabetes, or derived from weight/height)
- More specific BMI groupings will likely be considered within the clustering subsets.
- Provide summary data to the steering committee regarding those who would be excluded should the BMI cutoff be changed to 20 or 25 kg/m^2
-

For peer review only - http://bmjopen.bmj.com/site/about/guidelines.xhtml

Current drugs:

- It will be permissible for those within the target population to be on one or two of the three target drug classes as excluding these people is likely to unfavourably skew the target population.
- It will also be permissible to be taking other glucose lowering drugs including insulin, metformin, sulfonylureas, thiazolidinediones, alpha glucosidase inhibitors.
- There will be no limits on non-diabetes drugs including antihypertensives, ACEi/ARB, statins or antiplatelets
- There will be a limit on high dose oral steroids- exclude if currently on ≥prednisolone 5mg or equivalent (BNF codes 1.5.2, 6.3.2, 10.1.2) as of 1/1/19

Renal function:

- Limit to those with eGFR >30 ml/min/1.73m^2 (derived CKD EPI variable from within Diabepi).
- Exclude if current renal replacement therapy (linked Renal Registry Data within Diabepi)

Cardiovascular disease/risk:

- There will be no limit on prior cardiovascular disease, including heart failure, or cardiovascular risk factors e.g., smoking, dyslipidaemia at this stage.
- These factors will be considered further in the subset clustering
- ICD 10 codes for CV disease include coronary disease, cerebrovascular ischaemic disease, unspecified cerebral infarction, unspecified atherosclerosis, and peripheral vascular disease. (I have excluded haemorrhagic stroke disease when specified)

Specific exclusions:

- Any type of diagnosed diabetes other than type 2 diabetes mellitus
- Admission with DKA in the last 10 years defined via ICD10 codes linked to SMR01 admission data (ICD10: E10.1, E11.1, E13.1, E14.1) Note E10.1 is type 1 with ketoacidosis but leave in as check in case of coding errors.
- Renal function: Most recent eGFR≤ 30 ml/min/1.73m^2
- Urinary tract infection: Exclude if hospitalisation for urinary tract infection/urinary sepsis in last 10 years defined via ICD10 codes linked to SMR01 admission data (IC10: N39.0)
- Fungal infections: Exclude if 3 or more prescriptions for anti-fungal medication (oral, pessary or topical >1% strength) within the preceding 3 years defined using BNF code 5.2 within linked prescribing data. Whilst this will not 100% identify genitourinary fungal infections vs other dermatological fungal infections, limiting to oral, pessary and higher strength topical treatments is likely to limit the overlap somewhat.
- Pancreatitis/Pancreatic Insufficiency: Exclude if previously admitted to hospital with pancreatitis or pancreatic insufficiency in last 10 years defined via ICD10 codes linked to SMR01 admission data (ICD10: K85.0, K85.1, K85.2, K85.3, K85.8, K85.9, K86.0, K86.1, K87.1, (B25.2, B26.3)) or prescription of Pancreatin/Creon supplements as of 1/1/19 defined by BNF code 1.9.4
- Gallstone disease: Exclude if hospitalised with cholelithiasis or cholecystitis disease in last 10 years defined via ICD10 codes linked to SMR01 admission data (ICD10: K80.0, K80.1, K80.2, K80.3, K80.4, K80.5, K80.8, K81.0, K81.1, K81.8, K81.9). If person has had subsequent cholecystectomy can be included (OPCS surgical codes J18.1, J18.2, J18.3, J18.4, J18.5, J18.8, J18.9).

For peer review only - http://bmjopen.bmj.com/site/about/guidelines.xhtml

- Inflammatory Bowel Disease: Exclude if hospital admission with inflammatory bowel disease (UC/Crohn's Disease/Unspecified non infective inflammatory bowel disease) in last 10 years defined via ICD10 codes linked to SMR01 admission data (ICD 10: K50.0 , K50.1, K50.8, K50.9, K51.0, K51.2, K51.3, K51.4, K51.5, K51.8, K51.9 , K52.0, K52.1, K52.2, K52.3, K52.8, K52.9) Also exclude if immunotherapy (Unable to find with 1.5.3 BNF code. Instead used drugnames from non-steroid drugs mentioned in "British Society of Gastroenterology consensus guidelines on the management of inflammatory bowel disease in adults" = MESALAZINE', 'AZATHIOPRINE', 'MERCAPTOPURINE', 'METHOTREXATE','INFLIXIMAB', 'ADALIMUMAB', 'GOLIMUMAB', 'VEDOLIZUMAB', 'TOFACITINIB', 'USTEKINUMAB') plus ≥ 1 outpatient appointment at Gastroenterology within past 3 years based on SMR00 coding (Specialty= A9, attendance status= 1 (seen)).
  Whilst this will not identify those with milder disease in the community, and may include people with other diagnoses in error, in practical terms it will likely identify and exclude those with more severe disease in whom incretin therapies would be contraindicated.
- Gastroparesis: Whilst we intended to exclude for history of gastroparesis, there is no ICD10 code specific enough for this therefore it was not possible on the available data.
- Recent diagnosis of cancer (based on record in the smr06 cancer register database in last 3 years).
- End of Life: Exclude, based on SMR01 data, if admission from or discharge to a hospice at any time (location code =62), admission under palliative care (spec=AM), admission reason palliative care or geriatric palliative care (admreas=1M/4B) or admission to palliative care facility (sigfac=1G), as treatment unlikely to be appropriate

Variables of interest within target population

Aggregate descriptive characteristics from the target population will be gathered to facilitate trial outcome calibration in the next stage of this project.

Table S1: Variables of interest within routine datasets

1. Age in years
2. Duration of diabetes in years
3. Sex
4. BMI in kg/m^2
5. Ethnicity/Race
6. Systolic blood pressure in mmHg
7. Diastolic blood pressure in mmHg
8. Smoking status (never, previously, currently)
9. Previous cardiovascular disease (ICD10: I20.0, I20.1, I20.8, I20.9, I21.0, I21.1, I21.2, I21.3, I21.4, I21.9, I22.0, I22.1, I22.8, I22.9, I23.0, I23.1, I23.2, I23.3, I23.4, I23.5, I23.6, I23.8, I24.0 I24.1, I24.8, I24.9, I25.0, I25.1, I25.2, I25.3, I25.4, I25.5, I25.6, I25.8, I25.9, I63.0, I63.1, I63.2, I63.3, I63.4, I63.5, I63.6, I63.8, I63.9, I64.0, I65.0 , I65.1, I65.2, I65.3, I65.8, I65.9, I66.0, I66.1, I66.2, I66.3, I66.4, I66.8, I66.9, I67.2, I67.8, I67.9, I69.3, I69.4, I69.8, I70.0, I70.1, I70.2, I70.8, I70.9 , I73.0, I73.1, I73.8, I73.9) (yes or no)
10. History of heart failure (ICD: I50.0, I50.1, I50.9 in SMR01 data and/or currently on furosemide or bumetanide) (yes or no)

For peer review only - http://bmjopen.bmj.com/site/about/guidelines.xhtml

11.	Current non-insulin glucose lowering agents (BNF codes: 6.1.2) (yes/no)
12.	Current Insulin (yes or no) (BNF codes: 6.1.1)
13.	HbA1c (mean of recent) in mmol/mol
14.	Mean of recent eGFR ml/min/1.73m^2 (CKD EPI)
15.	Urine albumin to creatinine ratio in mg/g
16.	Total cholesterol in mmol/l
17.	Low Density Lipoprotein (LDL） in mmol/l
18.	High Density Lipoprotein (HDL) in mmol/l

Section 3: Statistical methods

Models will be fitted using the multilevel network meta-regression framework described by Phillippo et al[2], which we outline here.

IPD studies provide outcomes y_{ijk} and a vector of covariates $\boldsymbol{x}_{ijk}$ for each individual i in study j receiving treatment k. The individual-level model for these data is:

$$y_{ijk} \sim \pi_{\text{Ind}}(\theta_{ijk})$$

$$g(\theta_{ijk}) = \eta_{jk}(\boldsymbol{x}_{ijk}) = \mu_j + \boldsymbol{x}_{ijk}^T(\boldsymbol{\beta}_1 + \boldsymbol{\beta}_{2,k}) + \gamma_k$$

where $\pi_{\text{Ind}}(\cdot)$ is a suitable likelihood distribution. $g(\cdot)$ a suitable link function, which transforms the expected outcome θ_{ijk} for an individual conditional on their covariates onto the linear predictor η_{ijk}. μ_j are study-specific intercepts, $\boldsymbol{\beta}_1$ and $\boldsymbol{\beta}_{2,k}$ correspond to the effects of covariates and covariate-treatment interactions respectively, and γ_k is the individual-level treatment effect of treatment k compared to a chosen network reference treatment 1.

Aggregate studies provide aggregate outcomes $y_{\bullet jk}$ on treatment k in study j, and a joint distribution for the covariates $f_{jk}(\boldsymbol{x})$. The aggregate-level model for these data is constructed by integrating the individual-level model over the population in each study:

$$y_{\bullet jk} \sim \pi_{\text{Agg}}(\theta_{\bullet jk})$$

$$\theta_{\bullet jk} = \int_{\mathfrak{X}} g^{-1}(\eta_{jk}(\boldsymbol{x}))\, f_{jk}(\boldsymbol{x})\, d\boldsymbol{x}$$

where $\pi_{\text{Agg}}(\cdot)$ is a suitable likelihood distribution, $\theta_{\bullet jk}$ is the expected outcome on treatment k in study j, and $\mathfrak{X}$ is the support of the covariates. The integral is evaluated using efficient quasi-Monte Carlo numerical integration, with a sample of S points $\tilde{\boldsymbol{x}}_{jk;s}$ from the joint distribution $f_{jk}(\boldsymbol{x})$:

$$\theta_{\bullet jk} \approx S^{-1} \sum_{\tilde{\boldsymbol{x}}_{jk;s}} g^{-1}(\eta_{jk}(\tilde{\boldsymbol{x}}_{jk;s})).$$

The joint distribution of covariates $f_{jk}(\boldsymbol{x})$ is rarely available directly from study publications; instead, marginal summaries are available (e.g. means and standard deviations, proportions). However, under assumptions about the forms of the marginal distributions and the correlation structure (for example based on those observed in the IPD studies), the full joint distribution can be reconstructed. In practice, results are seen to be robust to misspecification of these assumptions[3].

For peer review only - http://bmjopen.bmj.com/site/about/guidelines.xhtml

In a Bayesian framework, prior distributions will be placed on each of the model parameters μ_j, $\boldsymbol{\beta}_1$, $\boldsymbol{\beta}_{2,k}$, γ_k. Random effects models and unrelated mean effects or node-splitting models will also be fitted within the above framework, to explore heterogeneity and inconsistency respectively.[2]

After model fitting, population-average treatment effects $d_{ab(P)}$ between any two treatments a and b, in a population P with mean covariate values $\bar{\boldsymbol{x}}_{(P)}$, can be obtained as

$$d_{ab(P)} = \bar{\boldsymbol{x}}_{(P)}^T(\boldsymbol{\beta}_{2,b} - \boldsymbol{\beta}_{2,a}) + \gamma_b - \gamma_a.$$

When outcomes are reported by subgroup in the aggregate studies, these can be incorporated by extending the aggregate-level model above as follows:

$$y_{\bullet jkl} = \pi_{\mathrm{Agg}}(\theta_{\bullet jkl})$$

$$\theta_{\bullet jkl} \approx S_{jkl}^{-1} \sum_{\tilde{\boldsymbol{x}}_{jkl;s}} g^{-1}(\eta_{jk}(\tilde{\boldsymbol{x}}_{jkl;s}))$$

where for each subgroup l the integration points from the full joint distribution are partitioned into each subgroup as $\tilde{\boldsymbol{x}}_{jkl;s}$. This approach is only appropriate for independent subgroups (e.g. levels of a single covariate, or subgroups of multiple covariates reported factorially). For non-independent subgroups (e.g. multiple single-covariate subgroup analyses), this approach will be extended to account for the resulting correlations in the likelihood.

References

1. Neal B, Perkovic V, de Zeeuw D, Mahaffey KW, Fulcher G, Stein P, et al. Rationale, design, and baseline characteristics of the Canagliflozin Cardiovascular Assessment Study (CANVAS)—A randomized placebo-controlled trial. Am Heart J. 2013 Aug 1;166(2):217-223.e11.

2. Phillippo DM, Dias S, Ades AE, Belger M, Brnabic A, Schacht A, et al. Multilevel network meta-regression for population-adjusted treatment comparisons. J R Stat Soc Ser A Stat Soc. 2020;183(3):1189–210.

3. Phillippo DM, Dias S, Ades AE, Welton NJ. Assessing the performance of population adjustment methods for anchored indirect comparisons: A simulation study. Stat Med. 2020;39(30):4885–911.

For peer review only

For peer review only - http://bmjopen.bmj.com/site/about/guidelines.xhtml

Reporting checklist for protocol of a systematic review and meta analysis.

Based on the PRISMA-P guidelines.

Instructions to authors

Complete this checklist by entering the page numbers from your manuscript where readers will find each of the items listed below.

Your article may not currently address all the items on the checklist. Please modify your text to include the missing information. If you are certain that an item does not apply, please write "n/a" and provide a short explanation.

Upload your completed checklist as an extra file when you submit to a journal.

In your methods section, say that you used the PRISMA-Preporting guidelines, and cite them as:

Moher D, Shamseer L, Clarke M, Ghersi D, Liberati A, Petticrew M, Shekelle P, Stewart LA. Preferred Reporting Items for Systematic Review and Meta-Analysis Protocols (PRISMA-P) 2015 statement. Syst Rev. 2015;4(1):1.

		Reporting Item	Page Number
**Title**			
Identification	#1a	Identify the report as a protocol of a systematic review	1
Update	#1b	If the protocol is for an update of a previous systematic review, identify as such	NA

For peer review only

**Registration**			
	#2	If registered, provide the name of the registry (such as PROSPERO) and registration number	3
**Authors**			
Contact	#3a	Provide name, institutional affiliation, e-mail address of all protocol authors; provide physical mailing address of corresponding author	1
Contribution	#3b	Describe contributions of protocol authors and identify the guarantor of the review	14
**Amendments**			
	#4	If the protocol represents an amendment of a previously completed or published protocol, identify as such and list changes; otherwise, state plan for documenting important protocol amendments	NA
**Support**			
Sources	#5a	Indicate sources of financial or other support for the review	14
Sponsor	#5b	Provide name for the review funder and / or sponsor	14
Role of sponsor or funder	#5c	Describe roles of funder(s), sponsor(s), and / or institution(s), if any, in developing the protocol	14
**Introduction**			
Rationale	#6	Describe the rationale for the review in the context of what is	4

For peer review only

		already known	
Objectives	#7	Provide an explicit statement of the question(s) the review will address with reference to participants, interventions, comparators, and outcomes (PICO)	5
**Methods**			
Eligibility criteria	#8	Specify the study characteristics (such as PICO, study design, setting, time frame) and report characteristics (such as years considered, language, publication status) to be used as criteria for eligibility for the review	6,7
Information sources	#9	Describe all intended information sources (such as electronic databases, contact with study authors, trial registers or other grey literature sources) with planned dates of coverage	7
Search strategy	#10	Present draft of search strategy to be used for at least one electronic database, including planned limits, such that it could be repeated	1,2 (SA)
Study records - data management	#11a	Describe the mechanism(s) that will be used to manage records and data throughout the review	7
Study records - selection process	#11b	State the process that will be used for selecting studies (such as two independent reviewers) through each phase of the review (that is, screening, eligibility and inclusion in meta-analysis)	7,8
Study records - data collection	#11c	Describe planned method of extracting data from reports (such as piloting forms, done independently, in duplicate), any	8

For peer review only

process		processes for obtaining and confirming data from investigators	
Data items	#12	List and define all variables for which data will be sought (such as PICO items, funding sources), any pre-planned data assumptions and simplifications	Table 1
Outcomes and prioritization	#13	List and define all outcomes for which data will be sought, including prioritization of main and additional outcomes, with rationale	6
Risk of bias in individual studies	#14	Describe anticipated methods for assessing risk of bias of individual studies, including whether this will be done at the outcome or study level, or both; state how this information will be used in data synthesis	9
Data synthesis	#15a	Describe criteria under which study data will be quantitatively synthesised	9,10
Data synthesis	#15b	If data are appropriate for quantitative synthesis, describe planned summary measures, methods of handling data and methods of combining data from studies, including any planned exploration of consistency (such as I2, Kendall's τ)	9,10
Data synthesis	#15c	Describe any proposed additional analyses (such as sensitivity or subgroup analyses, meta-regression)	9,10
Data synthesis	#15d	If quantitative synthesis is not appropriate, describe the type of summary planned	NA
Meta-bias(es)	#16	Specify any planned assessment of meta-bias(es) (such as publication bias across studies, selective reporting within	NA

For peer review only

		studies)	
Confidence in cumulative evidence	#17	Describe how the strength of the body of evidence will be assessed (such as GRADE)	NA

The PRISMA-P elaboration and explanation paper is distributed under the terms of the Creative Commons Attribution License CC-BY. This checklist was completed on 29. June 2022 using https://www.goodreports.org/, a tool made by the EQUATOR Network in collaboration with Penelope.ai

For peer review only

BMJ Open

Calibrating a network meta-analysis of diabetes trials of sodium glucose co-transporter 2 inhibitors, glucagon-like peptide-1 receptor analogues and dipeptidyl peptidase-4 inhibitors to a representative routine population: a systematic review protocol

Journal:	*BMJ Open*
Manuscript ID	bmjopen-2022-066491.R1
Article Type:	Protocol
Date Submitted by the Author:	13-Sep-2022
Complete List of Authors:	Butterly, Elaine; University of Glasgow Institute of Health and Wellbeing,<br>Wei, Lili; University of Glasgow Institute of Health and Wellbeing<br>Adler, Amanda ; University of Oxford,<br>Almazam, Saleh; University of Glasgow Institute of Health and Wellbeing<br>Alsallumi, Khalid; University of Glasgow Institute of Health and Wellbeing<br>Blackbourn, Luke; University of Edinburgh<br>Dias, Sofia; University of York, Centre for Reviews and Dissemination<br>Hanlon, Peter; University of Glasgow Institute of Health and Wellbeing<br>Hughes, Katherine; NHS Greater Glasgow and Clyde, Department of Diabetes, Glasgow Royal Infirmary<br>Lewsey, Jim; University of Glasgow Institute of Health and Wellbeing<br>Lindsay, Robert; University of Glasgow BHF Glasgow Cardiovascular Research Centre<br>McGurnaghan, Stuart; University of Edinburgh, MRC Institute of Genetics and Cancer<br>Petrie, John; University of Glasgow BHF Glasgow Cardiovascular Research Centre<br>Phillippo, David; University of Bristol<br>Sattar, Naveed; University of Glasgow, Institute of Cardiovascular and Medical Sciences<br>Tomlinson, Laurie; London School of Hygiene and Tropical Medicine, Epidemiology and Population Health<br>Welton, Nicky; University of Bristol<br>Wild, Sarah; University of Edinburgh, Public Health Sciences<br>McAllister, David; University of Glasgow Institute of Health and Wellbeing
**Primary Subject Heading**:	Epidemiology
Secondary Subject Heading:	Diabetes and endocrinology, Research methods
Keywords:	Clinical trials < THERAPEUTICS, DIABETES & ENDOCRINOLOGY, EPIDEMIOLOGY, PUBLIC HEALTH

SCHOLARONE™ Manuscripts

Title

Calibrating a network meta-analysis of diabetes trials of sodium glucose co-transporter 2 inhibitors, glucagon-like peptide-1 receptor analogues and dipeptidyl peptidase-4 inhibitors to a representative routine population: a systematic review protocol

Authors

Butterly EW[1], Wei L[1], Adler AI[2], Almazam SA[1], Alsallumi K[1], Blackbourn L[3], Dias S[4], Hanlon P[1], Hughes KA[5], Lewsey J[1], Lindsay RS[1], McGurnaghan S[3], Petrie JR[1], Phillippo DM[6], Sattar N[1], Tomlinson L[7], Welton NJ[6], Wild SH[3] and McAllister DA[1].

Affiliations

1 University of Glasgow, UK

2 Diabetes Trials Unit, University of Oxford, UK

3 University of Edinburgh, UK

4 Centre for Reviews and Dissemination, University of York, UK

5 Dept of Diabetes, Glasgow Royal Infirmary, UK

6 Population Health Sciences, Bristol Medical School, University of Bristol, UK

7 London School of Hygiene and Tropical Medicine, UK

Corresponding author

Professor David A McAllister

Wellcome Trust Intermediate Clinical Fellow, Beit Fellow, Professor of Clinical Epidemiology and Medical Informatics and Consultant in Public Health Medicine

School of Health and Wellbeing, University of Glasgow

1 Lilybank Gardens,

Glasgow G12 8RZ

Email: david.mcallister@glasgow.ac.uk

For peer review only

For peer review only - http://bmjopen.bmj.com/site/about/guidelines.xhtml

Abstract (300 words)

Introduction

Participants in randomised controlled trials (trials) are generally younger and healthier than many individuals encountered in clinical practice. Consequently, the applicability of trial findings is often uncertain. To address this, results from trials can be calibrated to more representative data sources. In a network meta-analysis, using a novel approach which allows the inclusion of trials whether or not individual-level participant data (IPD) is available, we will calibrate trials for three drug classes (sodium glucose co-transporter 2 (SGLT2) inhibitors, glucagon-like peptide-1 (GLP1) receptor analogues and dipeptidyl peptidase-4 (DPP4) inhibitors) to the Scottish diabetes register.

Methods and analysis

Medline and EMBASE databases, the US clinical trials registry (clinicaltrials.gov) and the Chinese Clinical Trial Registry (chictr.org.cn) will be searched from 1st January 2002. Two independent reviewers will apply eligibility criteria to identify trials for inclusion. Included trials will be phase 3 or 4 trials of SGLT2 inhibitors, GLP1 receptor analogues or DPP4 inhibitors, with placebo or active comparators, in participants with type 2 diabetes, with at least one of glycaemic control, change in body weight or major adverse cardiovascular event as outcomes. Unregistered trials will be excluded.

We have identified a target population from the population-based Scottish diabetes register. The chosen cohort comprises people in Scotland with type 2 diabetes who either 1) require further treatment due to poor glycaemic control where any of the three drug classes may be suitable, or 2) who have adequate glycaemic control but are already on one of the three drug classes of interest or insulin.

Ethics and dissemination

Ethical approval for IPD use was obtained from the University of Glasgow MVLS College Ethics Committee (Project: 200160070). The Scottish diabetes register has approval from the Scottish A Research Ethics Committee (11/AL/0225) and operates with Public Benefit and Privacy Panel for Health and Social Care approval (1617-0147).

Registration

This systematic review and meta-analysis is registered on PROSPERO (CRD42020184174)

For peer review only

For peer review only - http://bmjopen.bmj.com/site/about/guidelines.xhtml

Strengths and limitations of this study

- Where many previous reviews have focussed on fewer drugs or outcomes, the criteria used in this systematic review are designed to provide a definitive collection of phase 3 and 4 clinical trials of newer glucose lowering drugs.
- The planned calibration methodology will retain the strength of trial data (not breaking randomisation) while improving representativeness using routine healthcare data and can be used to calibrate trials to any target population of interest.
- Unlike other approaches to calibration the planned approach allows more studies to be included in the analysis due to the inclusion of both IPD and aggregate-level trials potentially reducing bias.
- Calibration modelling requires important assumptions, albeit fewer assumptions than simple extrapolation of trial results to wider populations.
- Calibration could produce misleading results if applied to populations who are entirely excluded from clinical trials, not just under-represented, or in the presence of additional modifiers of treatment effect not included in the model.

Keywords

Clinical trials, generalisability, representativeness, calibration, diabetes

Word count (excluding abstract)

4516

For peer review only

For peer review only - http://bmjopen.bmj.com/site/about/guidelines.xhtml

Introduction

Randomised controlled trials (hereafter abbreviated to trials) are the gold standard for obtaining unbiased estimates of treatment effects. However, trials are limited in terms of representativeness. Trial participants are on average younger, fitter and have fewer comorbid diseases than patients in routine care identified through disease registers, who are the target population for an intervention.[1–5]

In type 2 diabetes, one of the commonest chronic diseases,[6] this gap in representativeness is particularly evident. For example, compared to the population-based Scottish diabetes register, trial participants are on average younger and women are under-represented.[1] This under-representation remains true for trials of the newer glucose-lowering agents (sodium glucose co-transporter 2 (SGLT2) inhibitors, glucagon-like peptide-1 (GLP1) receptor analogues and dipeptidyl peptidase-4 (DPP4) inhibitors). [1,7–11] This problem of under-representation is acknowledged in clinical guidelines, for example the most recent diabetes guidance from the National Institute for Health and Care Excellence (NICE) states "much of the evidence base used to inform this guideline has been generated from studies involving younger adults (study mean ages ranged from 45 to 68 years)".[6]

However, the clinical implications of this under-representation are not self-evident. Since diabetes complications are commoner in older age groups,[12–14] people in routine care settings may benefit more from treatments than trial participants. Alternatively, since the risk of non-cardiovascular non-diabetes related deaths also increase with age, acting as a competing risk, routine care patients may benefit less than trial participants.[14] Therefore, in routine care settings, the applicability of trial results is uncertain.

Calibrating trial results to make them more representative of target populations in clinical practice is a promising approach to help address this uncertainty. First described by Cole and Stuart,[2] calibration involves re-analysing trial data using the prevalence of baseline characteristics in trial and target populations. Briefly, participants who, compared to the target population, are overrepresented in the trial (e.g. younger people) contribute less to the calibrated treatment effect estimate, while participants who are underrepresented (e.g. older people) contribute more. At the expense of wider confidence intervals for calibrated effects, this "moves" trials in the direction of increased representativeness. Most approaches to calibration respect randomisation and so avoid the confounding by indication which can occur when estimating treatment effects using observational data which is representative of the target population. Calibration also involves fewer assumptions than simpler approaches to extrapolating trial results to target populations.

For peer review only - http://bmjopen.bmj.com/site/about/guidelines.xhtml

Despite these advantages, calibration has not been widely used. Until recently, calibration required individual-level participant data (IPD) (or stratification of results for all levels and every combination of baseline characteristics) for all relevant trials, making it unfeasible in most settings. We propose to overcome this problem by using a novel calibration methodology which incorporates trials where IPD are available and trials where only published summary data are available in a single model. We will use this method to compare SGLT2 inhibitors, GLP1 receptor analogues and DPP4 inhibitors in type 2 diabetes.

Aim

To compare the efficacy of SGLT2 inhibitors, GLP1 receptor analogues and DPP4 inhibitors on glycated haemoglobin (HbA1c), body weight and cardiovascular outcomes in people with established type 2 diabetes by applying a network meta-analysis (NMA) of all relevant type 2 diabetes trials and calibrating to a selected target population from the Scottish diabetes register.

Objectives

1. To compare the efficacy of each drug class as an add on to metformin (dual therapy)
2. To compare the efficacy of each drug class as an add on to metformin plus one other glucose-lowering drug (triple therapy)
3. To compare the efficacy of each drug class singly (monotherapy)

As well as the NMA calibrated to the routine care population, to quantify the impact of the calibration on the final results we will also repeat 1-3 using an uncalibrated NMA and a NMA calibrated to the average population of the trials.

Methods

We plan to conduct a systematic review and calibrated NMA combining results from a model fitted to randomised controlled trials of the relevant drug classes (both IPD and aggregate-level data) with data for a target population defined using the Scottish diabetes register (Figure 1). The start and planned end dates of the study are as follows: 29/11/19 to 01/11/22. Here we describe the planned systematic review, planned modelling and the characterisation of the target population.

For peer review only - http://bmjopen.bmj.com/site/about/guidelines.xhtml

Systematic review

Eligibility criteria

This review will be performed in keeping with the Preferred Reporting Items for Systematic Reviews and Meta-Analyses statement (PRISMA) statement[15]. We used the PRISMA-P checklist for this protocol.[16] This review has been registered on PROSPERO (CRD42020184174).

Population

Eligible trials will study people over 18 years old with established type 2 diabetes, with trials of healthy volunteers, people with pre-diabetes or trials that include any other forms of diabetes e.g. type 1 diabetes or gestational diabetes, excluded. There will be no limit placed on duration or severity of diabetes and trials will be included if they examine a sub-population of people with type 2 diabetes defined by a comorbid condition e.g. a trial in people with type 2 diabetes and comorbid fatty liver disease.

Interventions

Eligible trials will study any SGLT2 inhibitor, GLP1 receptor analogue or DPP4 inhibitor as the intervention drug. This will not be limited to drugs approved by regulatory authorities in any specific country. Intervention drug preparations can be short acting or modified release and can be prescribed as mono-, dual- or triple therapy with other glucose lowering drugs including the other two classes of interest plus metformin, sulfonylureas, thiazolidinediones and insulin. Trials will be excluded when the intervention drug was given as a single dose only e.g. peri-operative trials of GLP1 receptor analogues. Trials will be excluded if they were performed under fasting conditions e.g. Ramadan specific trials, as this is likely to have influence on the treatment effect.

Comparators

Trials will be included where the intervention drug was compared to placebo or to an active pharmacological comparator e.g. metformin, sulfonylureas, thiazolidinediones, alpha-glucosidase inhibitors, SGLT2 inhibitors, GLP1 receptor analogues, DPP4 inhibitors, or insulins. Trials will be excluded if they had surgical comparators (e.g. bariatric surgery) or were compared to specific non-pharmacological lifestyle interventions (e.g. very low calorie diets).

Outcomes

The trial outcomes to be analysed are:

1. Glycaemic control- measured by change in glycated haemoglobin (HbA1c) in either % or mmol/mol

For peer review only

For peer review only - http://bmjopen.bmj.com/site/about/guidelines.xhtml

2. Change in body weight- measured by weight in kilograms or change in body mass index (BMI)
3. Cardiovascular outcome- measured as composite outcome such as Major Adverse Cardiovascular Events (MACE)

Trials will be included if at least one of the above outcomes was measured. It is not required to be the primary outcome of the trial. Non- inferiority trials will be included.

Study design

Eligible trials will be limited to randomised phase 3 or 4 trials. Trial registration is required as a marker of trial quality, but no specific registration platform has to be used. There will be no limitation based on trial blinding or enrolment size. Trials of any follow up length will be included except in cases where only HbA1c is reported, in which case these trials will only be included if the follow up length is ≥12 weeks from randomisation reflecting the physiological turnover of red blood cells. Exploratory sub-studies within a trial population e.g. where a small proportion of participants had an additional alternative intervention or exploratory outcome analysed, will be excluded. Other study designs including non-randomised and observational, along with existing meta-analyses, will be excluded.

Information sources

Relevant trials will be identified by systematic searches of Medline and EMBASE (via OVID) databases using a combination of Medical Subject Headings (MeSH) [17]and keyword searches. Terms will be piloted and refined then adapted to each database. (See supplementary appendix for full search strategy)

All searches will be limited from 1st January 2002, as pilot work showed the first phase 3 trials of relevant newer glucose-lowering drugs were all commenced after this date.

To reduce the risk of publication bias, two clinical trials registries (the US clinical trials registry at https://clinicaltrials.gov/[18] and the Chinese Clinical Trial Registry at https://www.chictr.org.cn/[19]) will also be searched for eligible trials using the same criteria.

Data management

The initial review stages will utilise Covidence online software [20] to manage the search records and the screening process. Eligible papers will be saved locally in pdf format and linked to the relevant trial via the corresponding trial registration identifier e.g. nctid. Data extracted from publicly available documents will be processed locally. Where IPD is available this will be processed on Vivli Center for Global Clinical Research Data[21] or Yale University Open Data Access Project (YODA)[22]

For peer review only - http://bmjopen.bmj.com/site/about/guidelines.xhtml

For peer review only

secure platforms and only approved aggregate level results will be exported and stored on csv files locally. At the time of publication, aggregate level data from the target population and trials will be made available along with sufficient metadata for analysis.

Selection process

Titles and abstracts obtained from the search strategies will be screened by one reviewer for potential relevance. Where the paper is potentially relevant (or if there is uncertainty), a full text paper will be acquired and reviewed in the next stage. A random sample of 100 titles and abstracts will be reviewed by an independent reviewer as a quality check. Two reviewers will then both independently review all full text papers and apply eligibility criteria. Reasons for excluding papers will be documented. Where there are conflicts, papers will be discussed in a meeting with at least two reviewers and a joint decision will be documented. Where required further information can be sought from sources such as trial registries to clarify if a paper contains a relevant trial. If a conflict of opinion remains, a third independent reviewer will be asked to review the paper. In the event an agreement cannot be made, the paper will be discussed with the steering committee for a final decision on inclusion. Papers will be included if they can be linked to a registered clinical trial meeting the eligibility criteria. The US or Chinese clinical trial registries will also be searched for relevant trials. The online registries will be searched by filtering trial condition as type 2 diabetes mellitus and trial product as any of the three classes of interest. The resulting trials will be screened for relevance using the same criteria used in the database searches. Included trials from the Medline and EMBASE searches, together with additional trials identified on the clinical trial registries, will be collated, and thereafter be identified by a unique trial registration identifier (e.g. a national clinical trial id (nctid) from clinicaltrials.gov).

Data collection process

Descriptive information for each trial (e.g. intervention class, follow up, enrolment) will be extracted from publicly available sources including online trial registries, published papers and study documents. Extraction of trial results will depend on the level of data accessible. The planned calibration analysis incorporates IPD and published trial-level aggregate data, depending on data availability. Two IPD repositories (Vivli and YODA) will be searched for data availability. Where IPD are not available, trial level aggregate data will be collected from publicly available sources. Where the trial is registered to the US Clinical Trial registry at ClinicalTrials.gov, this can be done semi-automatically by interrogation of the Aggregate Analysis of ClinicalTrials.gov (AACT) database[23]. Results extracted from AACT, and any code used, will be checked for accuracy by a second

For peer review only - http://bmjopen.bmj.com/site/about/guidelines.xhtml

researcher. Where trial results are not available on AACT, they will be double extracted from published documents manually by two researchers.

Data items

Individual level data/Aggregate level data		Trial descriptive data
**Baseline characteristics/ target population characteristics**	**Trial Outcomes**	**Trial Details**
• Age in years<br>• Sex<br>• Race/Ethnicity<br>• Geographical location<br>• Duration of type 2 diabetes mellitus in years<br>• Known cardiovascular disease- does the participant have history of atherosclerotic cardiovascular disease (coronary, cerebrovascular or peripheral vascular disease- see ICD10 codes below) (y/n)<br>• Use of any concomitant glucose lowering agents at baseline (y/n)<br>• Use of metformin at baseline (y/n)<br>• Use of sulphonylurea at baseline (y/n)<br>• Use of thiazolidinediones at baseline (y/n)<br>• Use of DPP4 inhibitor at baseline (y/n)<br>• Use of GLP1 receptor agonist at baseline (y/n)<br>• Use of SGLT2 inhibitor at baseline (y/n)	• HbA1c (% or mmol/mol)<br>• Body mass index (kg/m2)<br>• Body weight and height (kg, m)<br>• Cardiovascular outcome: MACE composite endpoint where available along with individual components (usually cardiovascular death, non-fatal myocardial infarction, and non-fatal stroke- but check study documents) and hospitalisation for heart failure where available<br>• Adverse events<br><br>**Note**: *All variables will be extracted in available units (which will be recorded) then can subsequently converted onto the desired scale.	• Trial identifier<br>• Trial title<br>• Brief description<br>• Phase<br>• Single vs multicentre trial<br>• Geographical location of trial<br>• Study design (e.g., blinded)<br>• Sponsor (e.g., Industry sponsor)<br>• Date of trial completion<br>Eligibility<br>• Inclusion/exclusion criteria<br>• Study enrolment number<br>Intervention<br>• Intervention drug (generic names)<br>• Comparator drug(s)/regimes<br>• Dosage and frequency of medications<br>• Other drugs allowed in arms<br>• Duration of follow up for each outcome

The data items to be extracted for each eligible trial are listed in Table 1. This will be individual level for the IPD trials and aggregate level data for the non-IPD trials. In trials where there is crossover or longer term follow up with escape treatment, data will be extracted for the initial randomised period only to reduce confounding by introduction of other agents. Baseline characteristics and outcomes will also be extracted for subgroup populations in trials reporting MACE where these are available.

Table 1: Data variables for extraction

For peer review only

For peer review only - http://bmjopen.bmj.com/site/about/guidelines.xhtml

• Use of insulin at baseline (y/n)<br>• Smoking status- Current smoker (y/n)<br>• eGFR (document what calculation used if this info is available)<br>• Serum creatinine (in case eGFR needs to be calculated)<br>• Urinary albumin creatinine ratio in mg/g<br>• Total/HDL/LDL Cholesterol in mmol/l<br>• Systolic blood pressure (mmHg)<br>• Diastolic blood pressure (mmHg)<br>• Body weight (kg)<br>• Height (m)	ICD10 codes to identify cardiovascular disease:<br>ICD10: I20.0, I20.1, I20.8, I20.9, I21.0, I21.1, I21.2, I21.3, I21.4, I21.9, I22.0, I22.1, I22.8, I22.9, I23.0, I23.1, I23.2, I23.3, I23.4, I23.5, I23.6, I23.8, I24.0 I24.1, I24.8, I24.9, I25.0, I25.1, I25.2, I25.3, I25.4, I25.5, I25.6, I25.8, I25.9, I63.0, I63.1, I63.2, I63.3, I63.4, I63.5, I63.6, I63.8, I63.9, I64.0, I65.0 , I65.1, I65.2, I65.3, I65.8, I65.9, I66.0, I66.1, I66.2, I66.3, I66.4, I66.8, I66.9, I67.2, I67.8, I67.9, I69.3, I69.4, I69.8, I70.0, I70.1, I70.2, I70.8, I70.9 , I73.0, I73.1, I73.8, I73.9	independently

Table 1: Data to be collected from routine data and trial data

Effect measures

For the IPD trials, participant level data will be re-analysed, and effect measures calculated. Outcome data from the intention to treat datasets will be extracted where available. For non-IPD trials, all available published measures will be extracted including arm level data (e.g. HbA1c at baseline and endpoint or change in HbA1c in each arm) and treatment effect estimates (e.g. ANCOVA). For cardiovascular outcome trials, count data or proportions for each arm will be extracted for the MACE outcome.

For peer review only - http://bmjopen.bmj.com/site/about/guidelines.xhtml

For peer review only

Risk of bias

Each eligible trial will be assessed for risk of bias using the Revised Cochrane risk-of-bias tool for randomized trials (RoB 2)[24] using two independent reviewers to assess risk of bias and provide detailed information on confidence of results. Where there are conflicts, papers will be discussed in a meeting with at least two reviewers and a joint decision will be documented. Where required further information can be sought from sources such as trial registries to clarify if a paper contains a relevant trial. If a conflict of opinion remains, a third independent reviewer will be asked to review the paper.

Calibrated NMA

We will perform calibrated NMA for the effect of treatment on glycaemic control, weight loss and cardiovascular outcome of glucose-lowering drugs from the three chosen classes. The formal details of the modelling– which is done within a Bayesian framework – are explained in detail in a separate publication [25], and the use of the modelling for this particular application is described in the supplementary appendix. Briefly, the modelling is conducted in two stages. First, a model is fitted to the trial IPD and trial aggregate level data to estimate treatment effects adjusting for differences in effect modifiers in the trial populations. Next, the fitted model is applied to the target population data to estimate treatment efficacy in that population.

For the main analysis, we will include the following covariates: age, duration of diabetes, HbA1c, estimated glomerular filtration rate (eGFR), total cholesterol, high density lipoprotein cholesterol, systolic blood pressure, diastolic blood pressure, BMI, sex, ethnicity, smoking status, history of cardiovascular disease, history of heart failure, metformin use and insulin use. For the routine data target population, we will use multiple imputation in order to account for missingness. We anticipate low missingness in the IPD therefore we will conduct a complete case analysis for the trial data. In sensitivity analyses we will examine the robustness of the findings to alternative covariate choices.

In the model fitting stage, there is a single underlying individual-level model across all studies, which includes terms for treatment effects, prognostic covariate effects, and treatment-covariate interactions (subgroup effects), with intercepts stratified by study terms for treatment effects and subgroup effects (t-c interactions). This is fitted directly to individuals in the IPD studies, and for the aggregate-level trials the individual-level model is integrated over the trial covariate distribution to obtain the aggregate-level model. Such integration requires information on the distribution of the covariates in each trial (i.e. the distribution of age, sex, BMI etc). Summary level information on the marginal covariate distributions is normally available in the table of baseline characteristics of

For peer review only - http://bmjopen.bmj.com/site/about/guidelines.xhtml

published trial manuscripts; and can be extracted as part of the systematic review and, together with estimated covariances from the IPD studies used to obtain the joint distributions of covariates for each trial. [25,26]

A trial with IPD contributes more information about treatment-covariate interactions than a trial with only aggregate-level data. Nonetheless, both IPD and aggregate-level trials contribute information to the estimation of such interactions. More importantly, the modelling approach enforces consistency for trials with both types of data, and assumes that the relative treatment effects are, conditional on the known covariate levels, similar across trials. This assumption is weaker than the standard assumption in NMA that relative treatment effects are *unconditionally* similar across trials.

If we assume that treatment-covariate interactions are equal (or exchangeable) for treatments within drug-classes, we can estimate these treatment-covariate interactions if there are one or more trials with IPD (or multiple aggregate-level trials across a range of covariate values) within each drug class. This assumption is also weaker than the standard assumption in NMA that there are no covariate-treatment interactions (at least for covariates not identically distributed in all trials).

In the second stage of calibration, the model results are applied to the target population data to estimate treatment efficacy in that population. This is achieved by integrating the estimated regression model for covariate-specific treatment effects over the joint covariate distribution in the target population to obtain population specific treatment effects. This can be done for any target population where information is available for the relevant covariates (or even for any hypothetical target population where an investigator is interested in the treatment effects for any given set of covariate levels).

In this calibrated NMA, for the MACE outcome only, we propose to extend the modelling by including subgroup-level effect estimates for those aggregate trials where subgroup-level data are available for the main covariates of interest. This will further improve precision and potentially allow for the equal interactions within classes assumption to be relaxed. The existing model framework allows inclusion of single subgroups (or fully factorial subgroups where effects are presented for all combinations of the covariates of interest). We will extend the modelling to the more usual scenario where potentially correlated sub-group effects are presented for multiple subgroups.

Target population

A key step in calibration is defining a specific target population of interest. For this research, the Scottish diabetes register has been used to identify the target population. The Scottish diabetes

For peer review only - http://bmjopen.bmj.com/site/about/guidelines.xhtml

register (SCI-diabetes) includes data from >99% of people in Scotland with a diagnosis of diabetes. The data in the Scottish diabetes register platform are extracted regularly from a national database that collates information relevant to diabetes from primary and secondary care that is linked to other datasets via deterministic linkage. Linked datasets include hospitalisations and deaths, prescribing and dispensing data and national renal and cancer registries providing a rich and representative dataset. For this calibration, the 2019 extract from SCI Diabetes was used. The target population comprises people in Scotland with type 2 diabetes who either 1) require further treatment due to inadequate glycaemic control (HbA1c ≥53mmol/mol) where any of the three drug classes may be suitable (i.e. no contraindications of any of the three classes and no alternative focus of treatment e.g. end of life), or 2) have adequate glycaemic control but are already on one of the three drug classes of interest or insulin. People with type 2 diabetes in Scotland alive on 1st January 2019 with diabetes duration of at least one year were first identified (n= 256,620). The cohort was then refined by applying eligibility criteria in a step wise fashion (Section 2, Supplementary Appendix). The criteria were agreed with the steering committee and will be finalised prior to any extraction of results from the clinical trial data. For some of the criteria, for example for body mass index and renal function, several cut-off options were examined to assess their impact on the final population characteristics, and the final decision chosen based on clinical judgement. For the clinical contraindications, diseases were identified via a combination of International Classification of Diseases (ICD) codes[27] from hospital admission data, prescribing data, and outpatient clinic attendance data. Additional details are provided in the supplementary appendix.

Subsequently, we will calibrate the trial results to specific sub-populations within the overall target population to estimate sub-population treatment effects. We will identify these sub-populations using clustering methods alongside clinical judgement and public engagement.

We described the impact of each step in the selection process on the population characteristics using means and standard deviations, medians and interquartile ranges and counts and percentages for symmetrically distributed continuous variables, skewed continuous variables and binary variables respectively.

Patient and public involvement

We have involved diabetes patient and public involvement (PPI) groups in the design and funding application stages of this work. The groups provided positive feedback and improved the readability of plain English summary of the study. We will continue to involve a local PPI group to help guide the subpopulations for calibration and to ensure any outputs are distributed in the best way to help people with diabetes. We have also invited people with diabetes to join our steering committee.

For peer review only - http://bmjopen.bmj.com/site/about/guidelines.xhtml

For peer review only

	Whole Scottish type 2 diabetes population ≥1 year post diagnosis	Defined Scottish Target Population for Calibration
n	256,620	127,992
n (%) male	144,338 (56.2%)	77,599 (60.6%)
Mean (SD) age in years	66.7 (12.7)	63.8 (12.1)
Mean (SD) body mass index in kg/m^2	31.7 (6.6)	32.7(6.2)
Mean (SD) duration diabetes in years	10.0 (7.1)	10.3 (6.9)
Mean (SD) HbA1c in mmol/mol; %	60.2 (15.1).<br>7.7% (1.7%)	67.4 (13.1).<br>8.3% (1.2%)
Mean (SD) estimated glomerular filtration rate in ml/min/1.73m^2	77.6 (21.3)	81.7 (19.9)

Results

Target population

We present the details of the target population chosen for calibration to ensure this is documented prior to any data analysis from the clinical trials. Initially, the whole population of people with type 2 diabetes in Scotland alive as of 1st January 2019 with a duration of diabetes of at least one year was identified (n=256,620). This cohort was 56.2% male with a mean age of 66.7 (standard deviation (SD), 12.7) years and had been diagnosed with type 2 diabetes for a mean (SD) of 10.0 (7.1) years. Mean (SD) BMI was 31.7 (6.6) kg/m^2, 14.5% were current smokers and 21.2% had a previous history of cardiovascular disease.

This whole population was then restricted to those defined as eligible for treatment escalation and in whom any of the three classes would be considered, to define the final target population for calibration (n= 127,992). This group was 60.6% male with a mean age of 63.8 (12.1) years and had been diagnosed with type 2 diabetes for a mean of 10.3 (6.9) years. Mean (SD) BMI was 32.7 (6.2) kg/m^2, 14.6% were current smokers and 17.6% had a previous history of cardiovascular disease. (Table 2)

Table 2 Summary characteristics of the Scottish target population

For peer review only - http://bmjopen.bmj.com/site/about/guidelines.xhtml

Mean (SD) total cholesterol in mmol/l	4.3 (1.0)	4.4 (1.0)
Mean (SD) high density lipoprotein cholesterol in mmol/l	1.2 (0.3)	1.1 (0.3)
Mean (SD) systolic blood pressure in mmHg	135.5 (12.2)	135.7 (11.9)
Mean (SD) diastolic blood pressure in mmHg	76.8 (7.6)	77.8 (7.6)
White racial group %	75.8%	75.6%
Asian racial group %	3.8%	4.2%
Black racial group %	0.5%	0.5%
Mixed or other racial group %	3.0%	3.1%
Race unknown %	17.0%	16.5%
% Current smokers	14.5%	14.6%
% History of heart failure	11.9%	9.1%
% History of cardiovascular disease	21.2%	17.6%
% Metformin use	55.2%	69.2%
% Insulin use	8.4%	10.3%

Table 2: Summary characteristics of the whole population within the Scottish diabetes register who have been diagnosed with type 2 diabetes for at least 1 year and a subset who are the defined target population. Missingness of these individual variables in the target population are as follows: sex (0%), age (0%), body mass index (3.4%), duration (0%), glycated haemoglobin (HbA1c) (1.2%), estimated glomerular filtration rate (1.5%), total cholesterol (2.1%), high density lipoprotein cholesterol (7.4%), systolic blood pressure (1.1%), diastolic blood pressure (1.1%), smoking status (7.4%)

Discussion

This study will use all the available IPD and aggregate-level trial data and data from a diabetes register to estimate the effectiveness of SGLT2 inhibitor, GLP1 receptor analogue and DPP4 inhibitors for patients in routine care settings. It will also be the first study, to our knowledge, to calibrate multiple trials (both IPD and aggregate level) to a representative target population defined using routinely collected healthcare data.

We have opted to perform calibrated NMA to address the differences in characteristics of people with type 2 diabetes in UK clinical practice and participants in trials for the included drug-classes.[1,7–11] A previous study, on applying trial eligibility for a major cardiovascular outcome trial of an SGLT2 inhibitor to a primary care database (n= 1,238,909), found that only 15.7% of people with type 2 diabetes had similar levels of cardiovascular risk to participants in the trial, and only 11.1% of SGLT2 inhibitor treated patients were comparable to the trial participants in terms of baseline

For peer review only - http://bmjopen.bmj.com/site/about/guidelines.xhtml

characteristics.[9] Similarly, a European study (n= 803, 836) of German, Norwegian, Swedish and Dutch populations found that the proportion who were eligible for the four main SGLT2 inhibitor cardiovascular outcome trials ranged from 17-59%.[10] Similarly, in the USA, a cross-sectional study evaluating the eligibility of people within the Diabetes Collaborative Registry (n=172,643) for SGLT2 inhibitor cardiovascular outcome trials reported that 48% of their population were ineligible for any one of the trials, with individual trial eligibility ranging from 26-44%. [8] However, while such studies quantified the degree of lack of representativeness, they do not allow us to assess the likely impact of such differences to the underrepresented populations.

An existing approach to address lack of representativeness is to estimate treatment effects using observational data. Known as pharmaco-epidemiology, such analyses commonly use routine healthcare data, where included individuals are more representative by definition. However, while such studies have mostly yielded results similar to those from comparable clinical trials,[28] they have led to inaccurate conclusions in several cases; this is because pharmaco-epidemiological analyses are limited by the problem of confounding by indication; treated and untreated patients differ in their susceptibility to disease-related outcomes and it is currently not possible to determine when such analyses have successfully overcome confounding by indication.[29–32]

Another existing approach commonly used in health technology assessments is to use simple extrapolation, wherein relative treatment effects from clinical trials are combined with data on event rates from other (ideally representative) data sources to estimate benefits and harms.[33] For example, on applying a relative risk of death of 0.80 from a clinical trial to a target population with one-year mortality of 10%, the expected absolute risk reduction for that target population is 2%. A strength of using simple extrapolation is that doing so avoids the problem of confounding by indication. However, in this approach one must assume that treatment effects are similar in trial and routine care populations regardless of differences in patient characteristics. This assumption can rarely be justified on biological or clinical grounds.

Trial calibration has the advantage over pharmaco-epidemiology that it avoids confounding by indication (as it does not break randomisation). However, it also has an advantage over simple extrapolation that it does not require the assumption that treatment effects are similar in trial and routine care settings, but only that treatment effects are similar for participants who are similar with respect to characteristics included in the modelling (e.g. with similar age, sex, BMI, etc). This assumption allows greater confidence in applying trial data to routine care settings suggesting that, alongside other methodologies (including pharmaco-epidemiology) it may have a valuable role in assessing the likely applicability of trial findings to participants in routine care settings.

For peer review only - http://bmjopen.bmj.com/site/about/guidelines.xhtml

The particular form of trial calibration we propose to use – calibrated NMA via multilevel network meta-regression– was recently developed to address limitations of conventional NMA (combining IPD and aggregate-level data in a coherent manner, whilst exploring and explaining heterogeneity in treatment effects according to differences in participant characteristics within and across trials) by co-authors (DP, NW, SD). We are not aware that this approach has previously been used to address the under-representation within clinical trials of participants with certain characteristics. This approach to calibrated NMA has a number of advantages. First, it allows us to calibrate trial findings to any target population, including sub-populations of clinical interest, provided one is willing to assume that treatment effects are similar for participants who are similar with respect to characteristics included in the modelling. Indeed, it even allows us to calibrate treatment effects to notional populations, allowing us to explore the sensitivity of trial findings under any plausible set of patient characteristics. Perhaps more importantly, the method allows us to use all relevant trials, whether or not IPD are available. This is likely to result in greater precision and less bias than methods which require access to individual-level participant data for all calibrated trials.

Limitations

Calibrated NMA is a potentially useful approach for assessing the applicability of trial findings to target populations in routine care settings, however, in addition to the assumptions stated above there are a number of limitations. First, if target populations and trial participants differ, even after conditioning on known characteristics, with respect to other characteristics which modify treatment effects, the calibrated treatment effects may be biased. Secondly, even where all important characteristics are included in the modelling, if they are incorrectly parameterised this may also cause misleading findings (e.g. if the true treatment-covariate interaction is non-linear and it is modelled with a linear term). Finally, some participants are entirely excluded from clinical trials, not simply under-represented (e.g. those with very severe frailty, extensive multimorbidity, or overwhelming personal circumstances such as severe dependence syndromes). While it may be technically possible to calibrate trial results to these groups (e.g. by extrapolation), findings from such analyses are likely to be misleading.

Ethics and dissemination

Ethical approval for use of individual participant level data was obtained from the University of Glasgow MVLS College Ethics Committee (Project No: 200160070). The Scottish diabetes register has approval from the Scottish A Research Ethics Committee (ref 11/AL/0225) and operates with Public Benefit and Privacy Panel for Health and Social Care approval (ref 1617-0147).

For peer review only - http://bmjopen.bmj.com/site/about/guidelines.xhtml

Data availability

The data required for this study will come from multiple sources. Published and publicly available trial data will be available from journal publications, clinical trial registries and published study documents. The IPD used will be available through application to third party repositories including Vivli Center for Global Clinical Research Data and Yale University Open Data Access Project (YODA). The data for target population calibration from the Scottish diabetes register is not publicly available. At the time of publication, aggregate level data from the target population and trials will be made available along with sufficient metadata for analysis.

Author contributions

DM, DP, SD and NW designed the study and EB and LW designed the search strategy. EB, and LW will conduct the systematic review screening. EB, LW, KA and SA will conduct the risk of bias scoring. LB and SM provided cleaned data and supported data acquisition in the Scottish diabetes register. EB conducted the analysis within the Scottish diabetes register, supervised by DM. EB and DM wrote the first draft of this protocol paper. AA, PH, KH, JL, RL, JP, NS, LT and SW are all expert members of the projects steering committee who have contributed to planning, design decisions requiring clinical input, and results interpretation from the Scottish diabetes register. AA, LB, PH, KH, JL, RL, SM, JP, NS, LT, SW, LW, SA, KA, SD, DP and NW reviewed, commented on, and made changes on the final protocol and manuscript.

Funding

This research is funded by the Medical Research Council (MR/T017112/1: Routine care treatment effectiveness in people with type 2 diabetes: Maximising the applicability of clinical trials)

David McAllister is funded via an Intermediate Clinical Fellowship and Beit Fellowship from the Wellcome Trust.

None of the funders had any influence over the study design, analysis, or decision to submit for publication.

Acknowledgements

We would like to acknowledge the people with diabetes in Scotland whose data make the diabetes register and the healthcare professionals who record these data while providing healthcare.

We would also like to thank the University of Glasgow Medical and Veterinary Life Sciences patient and public involvement and engagement group for their input regarding this study.

We would finally like to acknowledge and thank Daniel Kavanagh, MBChB student at the University of Glasgow, for his involvement in the early screening stages of this review.

For peer review only - http://bmjopen.bmj.com/site/about/guidelines.xhtml

Conflict of interests

There are no conflicts of interest to declare.

References

1. Saunders C, Byrne CD, Guthrie B, Lindsay RS, McKnight JA, Philip S, et al. External validity of randomized controlled trials of glycaemic control and vascular disease: how representative are participants? Diabet Med J Br Diabet Assoc. 2013 Mar;30(3):300–8.

2. Cole SR, Stuart EA. Generalizing evidence from randomized clinical trials to target populations: The ACTG 320 trial. Am J Epidemiol. 2010 Jul 1;172(1):107–15.

3. Coca SG, Krumholz HM, Garg AX, Parikh CR. Underrepresentation of renal disease in randomized controlled trials of cardiovascular disease. JAMA. 2006 Sep 20;296(11):1377–84.

4. Brown N, Melville M, Gray D, Young T, Skene AM, Wilcox RG, et al. Relevance of clinical trial results in myocardial infarction to medical practice: comparison of four year outcome in participants of a thrombolytic trial, patients receiving routine thrombolysis, and those deemed ineligible for thrombolysis. Heart Br Card Soc. 1999 Jun;81(6):598–602.

5. Udell JA, Wang TY, Li S, Kohli P, Roe MT, de Lemos JA, et al. Clinical Trial Participation After Myocardial Infarction in a National Cardiovascular Data Registry. JAMA. 2014 Aug 27;312(8):841–3.

6. Type 2 diabetes in adults: management | Guidance and guidelines | NICE [Internet]. [cited 2019 Feb 22]. Available from: https://www.nice.org.uk/guidance/ng28

7. Pintat S, Fenici P, Hammar N, Ji L, Khunti K, Medina J, et al. Eligibility of patients with type 2 diabetes for sodium–glucose cotransporter 2 inhibitor cardiovascular outcomes trials: a global perspective from the DISCOVER study. BMJ Open Diabetes Res Care. 2019 Mar 1;7(1):e000627.

8. Wittbrodt E, Chamberlain D, Arnold SV, Tang F, Kosiborod M. Eligibility of patients with type 2 diabetes for sodium-glucose co-transporter-2 inhibitor cardiovascular outcomes trials: An assessment using the Diabetes Collaborative Registry. Diabetes Obes Metab. 2019;21(8):1985–9.

9. McGovern A, Feher M, Munro N, de Lusignan S. Sodium-Glucose Co-transporter 2 (SGLT2) Inhibitor: Comparing Trial Data and Real-World Use. Diabetes Ther Res Treat Educ Diabetes Relat Disord. 2017 Apr;8(2):365–76.

10. Birkeland KI, Jørgensen ME, Carstensen B, Persson F, Gulseth HL, Thuresson M, et al. Cardiovascular mortality and morbidity in patients with type 2 diabetes following initiation of sodium-glucose co-transporter-2 inhibitors versus other glucose-lowering drugs (CVD-REAL Nordic): a multinational observational analysis. Lancet Diabetes Endocrinol. 2017 Sep 1;5(9):709–17.

11. Weng. A Distribution-based Method for Assessing The Differences between Clinical Trial Target Populations and Patient Populations in Electronic Health Records. Appl Clin Inform. 2014;5:463–479.

For peer review only

12. Read SH, McAllister DA, Colhoun HM, Farran B, Fischbacher C, Kerssens JJ, et al. Incident ischaemic stroke and Type 2 diabetes: trends in incidence and case fatality in Scotland 2004-2013. Diabet Med J Br Diabet Assoc. 2018;35(1):99–106.

13. Read SH, Kerssens JJ, McAllister DA, Colhoun HM, Fischbacher CM, Lindsay RS, et al. Trends in type 2 diabetes incidence and mortality in Scotland between 2004 and 2013. Diabetologia. 2016;59(10):2106–13.

14. Tancredi M, Rosengren A, Svensson AM, Kosiborod M, Pivodic A, Gudbjörnsdottir S, et al. Excess Mortality among Persons with Type 2 Diabetes. N Engl J Med. 2015 Oct 29;373(18):1720–32.

15. The PRISMA 2020 statement: an updated guideline for reporting systematic reviews | The BMJ [Internet]. [cited 2022 May 16]. Available from: https://www.bmj.com/content/372/bmj.n71

16. Moher D, Shamseer L, Clarke M, Ghersi D, Liberati A, Petticrew M, et al. Preferred reporting items for systematic review and meta-analysis protocols (PRISMA-P) 2015 statement. Syst Rev. 2015 Jan 1;4(1):1.

17. Home - MeSH - NCBI [Internet]. [cited 2022 May 16]. Available from: https://www.ncbi.nlm.nih.gov/mesh/

18. Home - ClinicalTrials.gov [Internet]. [cited 2020 Jul 1]. Available from: https://clinicaltrials.gov/

19. Chinese Clinical Trial Register (ChiCTR) - The world health organization international clinical trials registered organization registered platform [Internet]. [cited 2020 Jul 1]. Available from: http://www.chictr.org.cn/abouten.aspx

20. Covidence - Better systematic review management [Internet]. Covidence. [cited 2022 May 16]. Available from: https://www.covidence.org/

21. Vivli - Center for Global Clinical Research Data [Internet]. [cited 2022 May 16]. Available from: https://vivli.org/

22. The YODA Project [Internet]. [cited 2022 May 16]. Available from: https://yoda.yale.edu/

23. AACT Database | Clinical Trials Transformation Initiative [Internet]. [cited 2022 May 16]. Available from: https://aact.ctti-clinicaltrials.org/

24. RoB 2: A revised Cochrane risk-of-bias tool for randomized trials | Cochrane Bias [Internet]. [cited 2022 May 16]. Available from: https://methods.cochrane.org/bias/resources/rob-2-revised-cochrane-risk-bias-tool-randomized-trials

25. Phillippo DM, Dias S, Ades AE, Belger M, Brnabic A, Schacht A, et al. Multilevel network meta-regression for population-adjusted treatment comparisons. J R Stat Soc Ser A Stat Soc. 2020;183(3):1189–210.

26. Phillippo DM, Dias S, Ades AE, Welton NJ. Assessing the performance of population adjustment methods for anchored indirect comparisons: A simulation study. Stat Med. 2020;39(30):4885–911.

27. ICD-10 Version:2019 [Internet]. [cited 2022 May 16]. Available from: https://icd.who.int/browse10/2019/en

For peer review only - http://bmjopen.bmj.com/site/about/guidelines.xhtml

28. Anglemyer A, Horvath HT, Bero L. Healthcare outcomes assessed with observational study designs compared with those assessed in randomized trials. Cochrane Database Syst Rev. 2014 Apr 29;(4):MR000034.

29. Bosco JLF, Silliman RA, Thwin SS, Geiger AM, Buist DSM, Prout MN, et al. A most stubborn bias: no adjustment method fully resolves confounding by indication in observational studies. J Clin Epidemiol. 2010 Jan;63(1):64–74.

30. Hue TF, Cummings SR, Cauley JA, Bauer DC, Ensrud KE, Barrett-Connor E, et al. Effect of Bisphosphonate Use on Risk of Postmenopausal Breast Cancer. JAMA Intern Med. 2014 Oct;174(10):1550–7.

31. Rossouw JE, Anderson GL, Prentice RL, LaCroix AZ, Kooperberg C, Stefanick ML, et al. Risks and benefits of estrogen plus progestin in healthy postmenopausal women: principal results From the Women's Health Initiative randomized controlled trial. JAMA. 2002 Jul 17;288(3):321–33.

32. Criner GJ, Connett JE, Aaron SD, Albert RK, Bailey WC, Casaburi R, et al. Simvastatin for the Prevention of Exacerbations in Moderate-to-Severe COPD. N Engl J Med. 2014 Jun 5;370(23):2201–10.

33. Dias S, Welton NJ, Sutton AJ, Ades AE. NICE DSU Technical Support Document 1: Introduction to Evidence Synthesis for Decision Making. National Institute for Health and Clinical Excellence; 2011. (NICE DSU Technical Support Document in Evidence Synthesis).

For peer review only

For peer review only - http://bmjopen.bmj.com/site/about/guidelines.xhtml

Figure legends

Figure 1: Overview of output process. Abbreviations: Individual level participant data (IPD), glycated haemoglobin (HbA1c), relative risk measures (RR), composite measure of major adverse cardiovascular events (MACE).

For peer review only

For peer review only - http://bmjopen.bmj.com/site/about/guidelines.xhtml

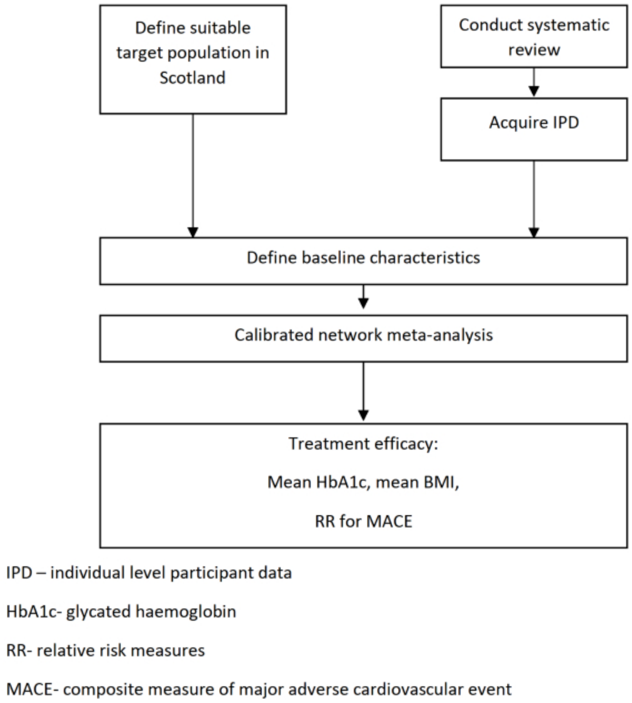

Figure 1: Overview of output process. Abbreviations: Individual level participant data (IPD), glycated haemoglobin (HbA1c), relative risk measures (RR), composite measure of major adverse cardiovascular events (MACE).

193x181mm (118 x 118 DPI)

Supplementary appendix

Section 1: Search strategies

Medline database

Table S1: Medline search strategy

# ▲	Searches	Results
1	non-insulin dependent diabetes mellitus/	125129
2	glucose intolerance/	8257
3	diabetic obesity/	0
4	impaired glucose tolerance/	8257
5	(non-insulin* depend* or noninsulin* depend* or noninsulindepend* or non insulindepend*).tw.	12075
6	((typ* 2 or typ* II) adj4 diabet*).tw.	132687
7	((adult* or matur* or late or slow or stabl* or obes*) adj4 diabet*).tw.	53350
8	(T2D* or DM2 or IIDM or MODY or NIDDM).tw.	35508
9	((nonketo* or non keto* or ketoresist* or keto resist*) adj4 diabet*).tw.	491
10	impaired glucose toleran*.tw.	10516
11	glucose intoleran*.tw.	10077
12	insulin* resistan*.tw.	76852
13	(insulin* defic* adj2 relativ*).tw.	184
14	(metabolic* syndrom* or plurimetabolic* syndrom*).tw.	46774
15	glucagon like peptide 1 receptor agonist/	0
16	(glucagon-like peptide 1 receptor inhibitor* or glucagon-like peptide 1 receptor agonist* or glucagon-like peptide 1 inhibitor* or glucagon-like peptide 1 agonist* or GLP-1 receptor inhibitor* or GLP-1 receptor agonist* or GLP-1 inhibitor* or GLP-1 agonist*).tw.	3107
17	albiglutide/	0
18	dulaglutide/	0
19	exendin 4/	2290
20	liraglutide/	1479
21	lixisenatide/	0
22	semaglutide/	0
23	taspoglutide/	0
24	albiglutide.tw.	168

For peer review only - http://bmjopen.bmj.com/site/about/guidelines.xhtml

25	dulaglutide.tw.	260
26	(exenatide or exendin 4).tw.	3063
27	liraglutide.tw.	2254
28	lixisenatide.tw.	342
29	semaglutide.tw.	254
30	taspoglutide.tw.	56
31	dipeptidyl peptidase IV inhibitor/	3609
32	(dipeptidyl-peptidase IV Inhibitor* or dipeptidyl-peptidase 4 Inhibitor* or ((DPP4 or DPP 4 or DPP IV) adj inhibitor*)).tw.	4570
33	alogliptin/	0
34	anagliptin/	0
35	gemigliptin/	0
36	linagliptin/	369
37	omarigliptin/	0
38	saxagliptin/	0
39	sitagliptin/	1300
40	teneligliptin/	0
41	vildagliptin/	592
42	alogliptin.tw.	419
43	anagliptin.tw.	59
44	gemigliptin.tw.	49
45	linagliptin.tw.	615
46	omarigliptin.tw.	37
47	saxagliptin.tw.	594
48	sitagliptin.tw.	2029
49	teneligliptin.tw.	116
50	vildagliptin.tw.	904
51	evogliptin.tw.	19
52	evogliptin/	0
53	sodium glucose cotransporter 2 inhibitor/	0
54	(sodium glucose transporter 2 inhibitor* or sodium glucose transporter ii inhibitor* or SGLT 2 inhibitor*).tw.	454
55	(sodium glucose cotransporter adj3 inhibitor*).tw.	1192
56	(sodium glucose co transporter adj3 inhibitor*).tw.	719
57	canagliflozin/	515
58	dapagliflozin/	0
59	empagliflozin/	0
60	ertugliflozin/	0
61	tofogliflozin/	0
62	canagliflozin.tw.	789
63	dapagliflozin.tw.	818
64	empagliflozin.tw.	862
65	ertugliflozin.tw.	61

For peer review only - http://bmjopen.bmj.com/site/about/guidelines.xhtml

66	tofogliflozin.tw.	86
67	ipragliflozin/	0
68	ipragliflozin.tw.	166
69	Randomized Controlled Trials as Topic/	126623
70	randomized controlled trial/	490217
71	Random Allocation/	100528
72	Double Blind Method/	153484
73	Single Blind Method/	27365
74	clinical trial/	518141
75	clinical trial, phase i.pt.	19368
76	clinical trial, phase ii.pt.	31271
77	clinical trial, phase iii.pt.	15567
78	clinical trial, phase iv.pt.	1754
79	controlled clinical trial.pt.	93274
80	randomized controlled trial.pt.	490217
81	multicenter study.pt.	257365
82	clinical trial.pt.	518141
83	exp Clinical Trials as topic/	330616
84	(clinical adj trial$).tw.	334395
85	((singl$ or doubl$ or treb$ or tripl$) adj (blind$3 or mask$3)).tw.	164050
86	PLACEBOS/	34468
87	placebo$.tw.	204278
88	randomly allocated.tw.	26477
89	(allocated adj2 random$).tw.	29629
90	or/69-89	1553327
91	or/1-14	295318
92	or/15-30	6907
93	or/31-52	7002
94	or/53-68	3313
95	92 or 93 or 94	15069
96	90 and 91 and 95	4292
97	case report.tw.	289645
98	letter/	1036792
99	historical article/	354203
100	or/97-99	1665591
101	96 not 100	4237
102	limit 101 to human	3612
103	limit 102 to english language	3452
104	limit 103 to yr="2002-Current"	3452

For peer review only - http://bmjopen.bmj.com/site/about/guidelines.xhtml

Embase database

Table S2: Embase search strategy

▲	Searches	Results
1	((diabetes or diabetes mellitus or diabetic*) adj1 (type 2 or type II or type ii or non-insulin dependent or noninsulin dependent or adult onset or mature onset or late onset)).tw	219042
2	(diabetic nephropath* or diabetic kidney disease).tw.	26837
3	glucose intolerance/	17965
4	diabetic obesity/	3737
5	impaired glucose tolerance/	30222
6	(non insulin* depend* or noninsulin* depend* or noninsulindepend* or non insulindepend*).tw.	14344
7	((typ* 2 or typ* II) adj4 diabet*).tw.	213585
8	((adult* or matur* or late or slow or stabl* or obes*) adj4 diabet*).tw.	85106
9	(T2D* or DM2 or IIDM or MODY or NIDDM).tw.	64908
10	((nonketo* or non keto* or ketoresist* or keto resist*) adj4 diabet*).tw.	721
11	impaired glucose toleran*.tw.	16435
12	glucose intoleran*.tw.	15372
13	insulin* resistan*.tw.	116387
14	(insulin* defic* adj2 relativ*).tw.	310
15	(metabolic* syndrom* or plurimetabolic* syndrom*).tw.	76726
16	glucagon like peptide 1 receptor agonist/	4508
17	(glucagon-like peptide 1 receptor inhibitor* or glucagon-like peptide 1 receptor agonist* or glucagon-like peptide 1 inhibitor* or glucagon-like peptide 1 agonist* or<br>GLP-1 receptor inhibitor* or GLP-1 receptor agonist* or GLP-1 inhibitor* or GLP-1 agonist*).tw.	5749
18	albiglutide/	871
19	dulaglutide/	1104
20	exendin 4/	9969
21	liraglutide/	7911
22	lixisenatide/	1317
23	semaglutide/	861
24	taspoglutide/	254
25	albiglutide.tw.	332
26	dulaglutide.tw.	654
27	(exenatide or exendin 4).tw.	5902
28	liraglutide.tw.	4839
29	lixisenatide.tw.	693
30	semaglutide.tw.	492
31	taspoglutide.tw.	110
32	dipeptidyl peptidase IV inhibitor/	8745
33	(dipeptidyl-peptidase IV Inhibitor* or dipeptidyl-peptidase 4 Inhibitor* or ((DPP4 or<br>DPP 4 or DPP IV) adj inhibitor*)).tw.	8196

For peer review only - http://bmjopen.bmj.com/site/about/guidelines.xhtml

34	alogliptin/	1740
35	anagliptin/	198
36	gemigliptin/	171
37	linagliptin/	2337
38	omarigliptin/	116
39	saxagliptin/	2941
40	sitagliptin/	7976
41	teneligliptin/	338
42	vildagliptin/	3759
43	alogliptin.tw.	739
44	anagliptin.tw.	122
45	gemigliptin.tw.	110
46	linagliptin.tw.	1304
47	omarigliptin.tw.	59
48	saxagliptin.tw.	1226
49	sitagliptin.tw.	4148
50	teneligliptin.tw.	238
51	vildagliptin.tw.	1744
52	evogliptin.tw.	37
53	evogliptin/	48
54	sodium glucose cotransporter 2 inhibitor/	3435
55	(sodium glucose transporter 2 inhibitor* or sodium glucose transporter ii inhibitor* or SGLT 2 inhibitor*).tw.	933
56	(sodium glucose cotransporter adj3 inhibitor*).tw.	1827
57	(sodium glucose co transporter adj3 inhibitor*).tw.	1295
58	canagliflozin/	2568
59	dapagliflozin/	2975
60	empagliflozin/	2877
61	ertugliflozin/	307
62	tofogliflozin/	286
63	canagliflozin.tw.	1536
64	dapagliflozin.tw.	1926
65	empagliflozin.tw.	1803
66	ertugliflozin.tw.	143
67	tofogliflozin.tw.	166
68	ipragliflozin/	484
69	ipragliflozin.tw.	284
70	or/1-15	455561
71	or/16-31	19351
72	or/32-53	19026
73	or/54-69	9181
74	71 or 72 or 73	38216
75	Clinical Trial/	984789
76	Randomized Controlled Trial/	576557

For peer review only

77	controlled clinical trial/	465986
78	multicenter study/	231443
79	Phase 3 clinical trial/	43062
80	Phase 4 clinical trial/	3641
81	exp RANDOMIZATION/	85032
82	Single Blind Procedure/	36861
83	Double Blind Procedure/	169262
84	Crossover Procedure/	61406
85	PLACEBO/	353947
86	randomi?ed controlled trial$.tw.	213219
87	rct.tw.	34296
88	(random$ adj2 allocat$).tw.	41310
89	single blind$.tw.	23926
90	double blind$.tw.	209043
91	((treble or triple) adj blind$).tw.	1070
92	placebo$.tw.	303630
93	Prospective Study/	557933
94	or/75-93	2246792
95	Case Study/	73901
96	case report.tw.	420554
97	abstract report/ or letter/	1124332
98	Conference proceeding.pt.	0
99	Conference abstract.pt.	3581657
100	Editorial.pt.	633720
101	Letter.pt.	1089718
102	Note.pt.	774711
103	or/95-102	6547596
104	94 not 103	1690232
105	70 and 74 and 104	6157
106	limit 105 to human	6042
107	limit 106 to english language	5816
108	limit 107 to yr="2002 -Current"	5812

For peer review only

For peer review only - http://bmjopen.bmj.com/site/about/guidelines.xhtml

Section 2: Protocol for routine healthcare data target population

Scope:

The scope of this document is to set out a protocol for identifying a clinically appropriate target population for calibration modelling within the routine datasets.

Aim:

1) To identify a clinically appropriate target population within the Scottish diabetes register for calibration modelling of a large network meta-analysis of glucose lowering drugs
2) Document the variables to be collected and summarised within the identified population

Background

For the proposed calibration modelling to be clinically relevant, the routine data target population to which the models are applied requires to be clearly set out and clinically justifiable. Using a 2019 extract of the SCI-diabetes database we aim to identify a population of people with type 2 diabetes mellitus where prescription of any of the three drug classes of interest (Sodium Glucose Co-Transporter 2 Inhibitors (SGLT2i) /Glucagon-Like Peptide 1 Receptor Agonists (GLP1ra) / Dipeptidyl Peptidase-4 Inhibitors (DPP4i)) would realistically be considered should the individual require treatment escalation. We aim to exclude anyone who would be considered to have a significant contraindication to any of the three drug classes.

Subsequent work will include clustering to identify more specific subsets of the population e.g., based on age, sex, body weight, renal function, cardiovascular risk. This will allow calibration to more specific subsets of the overall target population. This will be described in a later document.

Pilot work

We conducted some exploratory searches of the 2017 extract of SCI-diabetes to help guide this protocol. We identified those within the register who were prescribed at least one of the drug classes of interest. Overall, we identified 56,867 people on at least one of the target drugs. (Mean age 64.65 years, weight 98.14kg, glycated haemoglobin (HbA1c) 66.97mmol/mol, systolic blood pressure 136.55mmHg, estimated glomerular filtration rate (eGFR) 58.45. ml/min/1.73m^2).

Proposed steps

1) Access the 2019 data extract- and familiarise with datasets available within and data included in each.
2) Limit included participants to those where absolute contraindications for proposed drug classes are absent (see specific exclusions).
3) Extract data on descriptive variables from Table 1 where available.
4) Continuous observations for each individual will be taken as the mean of measurements over 3 years prior to 1/1/19. The most recent measurement in last 3 years will be taken for categorical variables e.g. smoking.
5) If all of the following variables are missing for the last 3 years, we will presume likely that the individual has either moved away or is not engaged with clinical services and they will not be included: HbA1c, systolic blood pressure, diastolic blood pressure, smoking status, fasting plasma glucose, urinary albumin creatinine ratio, total cholesterol, high density lipoprotein cholesterol, low density lipoprotein cholesterol, eGFR and body mass index.
6) Previous comorbidities/prescriptions will be extracted as present if appear in previous 10 years of data.
7) Comorbidity data will be defined using ICD10 codes within the linked SMR01 dataset (specified below). As per large cardiovascular outcome trials e.g. CANVAS[1], history of

For peer review only - http://bmjopen.bmj.com/site/about/guidelines.xhtml

cardiovascular disease will be defined as history of atherosclerotic cardiovascular disease including coronary, cerebrovascular or peripheral vascular disease.

8) Comorbidity data from SMR01/prescribing data will be included where the comorbidity appears in any position in the discharge data e.g., primary diagnosis or any other position of diagnosis
9) Create preliminary definition of overall population to be used for calibration based on above which may include modification of variables collected based on availability.
10) Provide summary statistics including number included/excluded to steering committee and (PRIOR to performing calibration or running NMA model on trial data) amend target population protocol on basis of feedback.

Proposed population defining characteristics

Timeframe:

- Date of diagnosis of type 2 diabetes mellitus at least one year prior to 1st Jan 2019
- Limit comorbidity data to a ten year look back
- Must be alive at time of extraction therefore exclude if death on or before chosen date (1st January 2019)

Age:

- Limit to 18 years old or above on 1st Jan 2019 or at diagnosis of diabetes
- No upper limits based on current age or age at diagnosis

Sex:

- No limits based on sex

Diagnosis:

- Must have documented diagnosis of type 2 diabetes mellitus within the derived diagnosis variable in dataset diagnosed before or on 1/1/18
- There will be no limits to the duration of diabetes diagnosis
- Those with diabetes in remission will be excluded when HbA1c limit applied.

Glycaemic control:

- No limit to HbA1c at diagnosis
- Limit population to those with most recent HbA1c ≥53mmol/mol or those with HbA1c <53mmol/mol but currently on one of the three drug classes of interest, or insulin.

Body Mass Index:

- Limit to those with most recent BMI measurement to ≥23.5kg/m^2 (use cleaned variable either from clinician entered variable from Sci Diabetes, or derived from weight/height)
- More specific BMI groupings will likely be considered within the clustering subsets.
- Provide summary data to the steering committee regarding those who would be excluded should the BMI cutoff be changed to 20 or 25 kg/m^2
-

Current drugs:

For peer review only - http://bmjopen.bmj.com/site/about/guidelines.xhtml

- It will be permissible for those within the target population to be on one or two of the three target drug classes as excluding these people is likely to unfavourably skew the target population.
- It will also be permissible to be taking other glucose lowering drugs including insulin, metformin, sulfonylureas, thiazolidinediones, alpha glucosidase inhibitors.
- There will be no limits on non-diabetes drugs including antihypertensives, ACEi/ARB, statins or antiplatelets
- There will be a limit on high dose oral steroids- exclude if currently on ≥prednisolone 5mg or equivalent (BNF codes 1.5.2, 6.3.2, 10.1.2) as of 1/1/19

Renal function:

- Limit to those with eGFR >30 ml/min/1.73m^2 (derived CKD EPI variable from within Diabepi).
- Exclude if current renal replacement therapy (linked Renal Registry Data within Diabepi)

Cardiovascular disease/risk:

- There will be no limit on prior cardiovascular disease, including heart failure, or cardiovascular risk factors e.g., smoking, dyslipidaemia at this stage.
- These factors will be considered further in the subset clustering
- ICD 10 codes for CV disease include coronary disease, cerebrovascular ischaemic disease, unspecified cerebral infarction, unspecified atherosclerosis, and peripheral vascular disease. (I have excluded haemorrhagic stroke disease when specified)

Specific exclusions:

- Any type of diagnosed diabetes other than type 2 diabetes mellitus
- Admission with DKA in the last 10 years defined via ICD10 codes linked to SMR01 admission data (ICD10: E10.1, E11.1, E13.1, E14.1) Note E10.1 is type 1 with ketoacidosis but leave in as check in case of coding errors.
- Renal function: Most recent eGFR≤ 30 ml/min/1.73m^2
- Urinary tract infection: Exclude if hospitalisation for urinary tract infection/urinary sepsis in last 10 years defined via ICD10 codes linked to SMR01 admission data (IC10: N39.0)
- Fungal infections: Exclude if 3 or more prescriptions for anti-fungal medication (oral, pessary or topical >1% strength) within the preceding 3 years defined using BNF code 5.2 within linked prescribing data. Whilst this will not 100% identify genitourinary fungal infections vs other dermatological fungal infections, limiting to oral, pessary and higher strength topical treatments is likely to limit the overlap somewhat.
- Pancreatitis/Pancreatic Insufficiency: Exclude if previously admitted to hospital with pancreatitis or pancreatic insufficiency in last 10 years defined via ICD10 codes linked to SMR01 admission data (ICD10: K85.0, K85.1, K85.2, K85.3, K85.8, K85.9, K86.0, K86.1, K87.1, (B25.2, B26.3)) or prescription of Pancreatin/Creon supplements as of 1/1/19 defined by BNF code 1.9.4
- Gallstone disease: Exclude if hospitalised with cholelithiasis or cholecystitis disease in last 10 years defined via ICD10 codes linked to SMR01 admission data (ICD10: K80.0, K80.1, K80.2, K80.3, K80.4, K80.5, K80.8, K81.0, K81.1, K81.8, K81.9). If person has had subsequent cholecystectomy can be included (OPCS surgical codes J18.1, J18.2, J18.3, J18.4, J18.5, J18.8, J18.9).
- Inflammatory Bowel Disease: Exclude if hospital admission with inflammatory bowel disease (UC/Crohn’s Disease/Unspecified non infective inflammatory bowel disease) in last 10 years

For peer review only - http://bmjopen.bmj.com/site/about/guidelines.xhtml

defined via ICD10 codes linked to SMR01 admission data (ICD 10: K50.0 , K50.1, K50.8, K50.9, K51.0, K51.2, K51.3, K51.4, K51.5, K51.8, K51.9 , K52.0, K52.1, K52.2, K52.3, K52.8, K52.9) Also exclude if immunotherapy (Unable to find with 1.5.3 BNF code. Instead used drugnames from non-steroid drugs mentioned in "British Society of Gastroenterology consensus guidelines on the management of inflammatory bowel disease in adults" = MESALAZINE', 'AZATHIOPRINE', 'MERCAPTOPURINE', 'METHOTREXATE','INFLIXIMAB', 'ADALIMUMAB', 'GOLIMUMAB', 'VEDOLIZUMAB', 'TOFACITINIB', 'USTEKINUMAB') plus ≥ 1 outpatient appointment at Gastroenterology within past 3 years based on SMR00 coding (Specialty= A9, attendance status= 1 (seen)).
Whilst this will not identify those with milder disease in the community, and may include people with other diagnoses in error, in practical terms it will likely identify and exclude those with more severe disease in whom incretin therapies would be contraindicated.

- Gastroparesis: Whilst we intended to exclude for history of gastroparesis, there is no ICD10 code specific enough for this therefore it was not possible on the available data.
- Recent diagnosis of cancer (based on record in the smr06 cancer register database in last 3 years).
- End of Life: Exclude, based on SMR01 data, if admission from or discharge to a hospice at any time (location code =62), admission under palliative care (spec=AM), admission reason palliative care or geriatric palliative care (admreas=1M/4B) or admission to palliative care facility (sigfac=1G), as treatment unlikely to be appropriate

Variables of interest within target population

Aggregate descriptive characteristics from the target population will be gathered to facilitate trial outcome calibration in the next stage of this project.

Table S1: Variables of interest within routine datasets

1. Age in years
2. Duration of diabetes in years
3. Sex
4. BMI in kg/m^2
5. Ethnicity/Race
6. Systolic blood pressure in mmHg
7. Diastolic blood pressure in mmHg
8. Smoking status (never, previously, currently)
9. Previous cardiovascular disease (ICD10: I20.0, I20.1, I20.8, I20.9, I21.0, I21.1, I21.2, I21.3, I21.4, I21.9, I22.0, I22.1, I22.8, I22.9, I23.0, I23.1, I23.2, I23.3, I23.4, I23.5, I23.6, I23.8, I24.0 I24.1, I24.8, I24.9, I25.0, I25.1, I25.2, I25.3, I25.4, I25.5, I25.6, I25.8, I25.9, I63.0, I63.1, I63.2, I63.3, I63.4, I63.5, I63.6, I63.8, I63.9, I64.0, I65.0 , I65.1, I65.2, I65.3, I65.8, I65.9, I66.0, I66.1, I66.2, I66.3, I66.4, I66.8, I66.9, I67.2, I67.8, I67.9, I69.3, I69.4, I69.8, I70.0, I70.1, I70.2, I70.8, I70.9 , I73.0, I73.1, I73.8, I73.9) (yes or no)
10. History of heart failure (ICD: I50.0, I50.1, I50.9 in SMR01 data and/or currently on furosemide or bumetanide) (yes or no)
11. Current non-insulin glucose lowering agents (BNF codes: 6.1.2) (yes/no)
12. Current Insulin (yes or no) (BNF codes: 6.1.1)
13. HbA1c (mean of recent) in mmol/mol

For peer review only - http://bmjopen.bmj.com/site/about/guidelines.xhtml

14.	Mean of recent eGFR ml/min/1.73m^2 (CKD EPI)
15.	Urine albumin to creatinine ratio in mg/g
16.	Total cholesterol in mmol/l
17.	Low Density Lipoprotein (LDL) in mmol/l
18.	High Density Lipoprotein (HDL) in mmol/l

Section 3: Statistical methods

Models will be fitted using the multilevel network meta-regression framework described by Phillippo et al[2], which we outline here.

IPD studies provide outcomes y_{ijk} and a vector of covariates $\boldsymbol{x}_{ijk}$ for each individual i in study j receiving treatment k. The individual-level model for these data is:

$$y_{ijk} \sim \pi_{\text{Ind}}(\theta_{ijk})$$

$$g(\theta_{ijk}) = \eta_{jk}(\boldsymbol{x}_{ijk}) = \mu_j + \boldsymbol{x}_{ijk}^T(\boldsymbol{\beta}_1 + \boldsymbol{\beta}_{2,k}) + \gamma_k$$

where $\pi_{\text{Ind}}(\cdot)$ is a suitable likelihood distribution. $g(\cdot)$ a suitable link function, which transforms the expected outcome θ_{ijk} for an individual conditional on their covariates onto the linear predictor η_{ijk}. μ_j are study-specific intercepts, $\boldsymbol{\beta}_1$ and $\boldsymbol{\beta}_{2,k}$ correspond to the effects of covariates and covariate-treatment interactions respectively, and γ_k is the individual-level treatment effect of treatment k compared to a chosen network reference treatment 1.

Aggregate studies provide aggregate outcomes $y_{\bullet jk}$ on treatment k in study j, and a joint distribution for the covariates $f_{jk}(\boldsymbol{x})$. The aggregate-level model for these data is constructed by integrating the individual-level model over the population in each study:

$$y_{\bullet jk} \sim \pi_{\text{Agg}}(\theta_{\bullet jk})$$

$$\theta_{\bullet jk} = \int_{\mathfrak{X}} g^{-1}\left(\eta_{jk}(\boldsymbol{x})\right) f_{jk}(\boldsymbol{x})\, d\boldsymbol{x}$$

where $\pi_{\text{Agg}}(\cdot)$ is a suitable likelihood distribution, $\theta_{\bullet jk}$ is the expected outcome on treatment k in study j, and $\mathfrak{X}$ is the support of the covariates. The integral is evaluated using efficient quasi-Monte Carlo numerical integration, with a sample of S points $\widetilde{\boldsymbol{x}}_{jk;s}$ from the joint distribution $f_{jk}(\boldsymbol{x})$:

$$\theta_{\bullet jk} \approx S^{-1} \sum_{\widetilde{\boldsymbol{x}}_{jk;s}} g^{-1}\left(\eta_{jk}(\widetilde{\boldsymbol{x}}_{jk;s})\right).$$

The joint distribution of covariates $f_{jk}(\boldsymbol{x})$ is rarely available directly from study publications; instead, marginal summaries are available (e.g. means and standard deviations, proportions). However, under assumptions about the forms of the marginal distributions and the correlation structure (for example based on those observed in the IPD studies), the full joint distribution can be reconstructed. In practice, results are seen to be robust to misspecification of these assumptions[3].

In a Bayesian framework, prior distributions will be placed on each of the model parameters μ_j, $\boldsymbol{\beta}_1$, $\boldsymbol{\beta}_{2,k}$, γ_k. Random effects models and unrelated mean effects or node-splitting models will also be fitted within the above framework, to explore heterogeneity and inconsistency respectively.[2]

After model fitting, population-average treatment effects $d_{ab(P)}$ between any two treatments a and b, in a population P with mean covariate values $\overline{\boldsymbol{x}}_{(P)}$, can be obtained as

For peer review only - http://bmjopen.bmj.com/site/about/guidelines.xhtml

$$d_{ab(P)} = \overline{\boldsymbol{x}}_{(P)}^{T}\left(\boldsymbol{\beta}_{2,b} - \boldsymbol{\beta}_{2,a}\right) + \gamma_b - \gamma_a.$$

When outcomes are reported by subgroup in the aggregate studies, these can be incorporated by extending the aggregate-level model above as follows:

$$y_{\bullet jkl} = \pi_{\text{Agg}}\left(\theta_{\bullet jkl}\right)$$

$$\theta_{\bullet jkl} \approx S_{jkl}^{-1} \sum_{\widetilde{\boldsymbol{x}}_{jkl;s}} g^{-1}\left(\eta_{jk}\left(\widetilde{\boldsymbol{x}}_{jkl;s}\right)\right)$$

where for each subgroup l the integration points from the full joint distribution are partitioned into each subgroup as $\widetilde{\boldsymbol{x}}_{jkl;s}$. This approach is only appropriate for independent subgroups (e.g. levels of a single covariate, or subgroups of multiple covariates reported factorially). For non-independent subgroups (e.g. multiple single-covariate subgroup analyses), this approach will be extended to account for the resulting correlations in the likelihood.

References

1. Neal B, Perkovic V, de Zeeuw D, Mahaffey KW, Fulcher G, Stein P, et al. Rationale, design, and baseline characteristics of the Canagliflozin Cardiovascular Assessment Study (CANVAS)—A randomized placebo-controlled trial. Am Heart J. 2013 Aug 1;166(2):217-223.e11.

2. Phillippo DM, Dias S, Ades AE, Belger M, Brnabic A, Schacht A, et al. Multilevel network meta-regression for population-adjusted treatment comparisons. J R Stat Soc Ser A Stat Soc. 2020;183(3):1189–210.

3. Phillippo DM, Dias S, Ades AE, Welton NJ. Assessing the performance of population adjustment methods for anchored indirect comparisons: A simulation study. Stat Med. 2020;39(30):4885–911.

For peer review only - http://bmjopen.bmj.com/site/about/guidelines.xhtml

For peer review only

Reporting checklist for protocol of a systematic review and meta analysis.

Based on the PRISMA-P guidelines.

Instructions to authors

Complete this checklist by entering the page numbers from your manuscript where readers will find each of the items listed below.

Your article may not currently address all the items on the checklist. Please modify your text to include the missing information. If you are certain that an item does not apply, please write "n/a" and provide a short explanation.

Upload your completed checklist as an extra file when you submit to a journal.

In your methods section, say that you used the PRISMA-Preporting guidelines, and cite them as:

Moher D, Shamseer L, Clarke M, Ghersi D, Liberati A, Petticrew M, Shekelle P, Stewart LA. Preferred Reporting Items for Systematic Review and Meta-Analysis Protocols (PRISMA-P) 2015 statement. Syst Rev. 2015;4(1):1.

		Reporting Item	Page Number
**Title**			
Identification	#1a	Identify the report as a protocol of a systematic review	1
Update	#1b	If the protocol is for an update of a previous systematic review, identify as such	NA
**Registration**			
	#2	If registered, provide the name of the registry (such as PROSPERO) and registration number	2
**Authors**			
Contact	#3a	Provide name, institutional affiliation, e-mail address of all protocol authors; provide physical mailing address of corresponding author	1

For peer review only

Contribution	#3b	Describe contributions of protocol authors and identify the guarantor of the review	18
**Amendments**			
	#4	If the protocol represents an amendment of a previously completed or published protocol, identify as such and list changes; otherwise, state plan for documenting important protocol amendments	NA
**Support**			
Sources	#5a	Indicate sources of financial or other support for the review	18/19
Sponsor	#5b	Provide name for the review funder and / or sponsor	18/19
Role of sponsor or funder	#5c	Describe roles of funder(s), sponsor(s), and / or institution(s), if any, in developing the protocol	18/19
**Introduction**			
Rationale	#6	Describe the rationale for the review in the context of what is already known	4
Objectives	#7	Provide an explicit statement of the question(s) the review will address with reference to participants, interventions, comparators, and outcomes (PICO)	5,6
**Methods**			
Eligibility criteria	#8	Specify the study characteristics (such as PICO, study design, setting, time frame) and report characteristics (such as years considered, language, publication status) to be used as criteria for eligibility for the review	6,7
Information sources	#9	Describe all intended information sources (such as electronic databases, contact with study authors, trial registers or other grey literature sources) with planned dates of coverage	7
Search strategy	#10	Present draft of search strategy to be used for at least one electronic database, including planned limits, such that it could be repeated	1,2 (SA)

For peer review only

Study records - data management	#11a	Describe the mechanism(s) that will be used to manage records and data throughout the review	7
Study records - selection process	#11b	State the process that will be used for selecting studies (such as two independent reviewers) through each phase of the review (that is, screening, eligibility and inclusion in meta-analysis)	7,8
Study records - data collection process	#11c	Describe planned method of extracting data from reports (such as piloting forms, done independently, in duplicate), any processes for obtaining and confirming data from investigators	8
Data items	#12	List and define all variables for which data will be sought (such as PICO items, funding sources), any pre-planned data assumptions and simplifications	9/10 Table 1
Outcomes and prioritization	#13	List and define all outcomes for which data will be sought, including prioritization of main and additional outcomes, with rationale	6
Risk of bias in individual studies	#14	Describe anticipated methods for assessing risk of bias of individual studies, including whether this will be done at the outcome or study level, or both; state how this information will be used in data synthesis	10
Data synthesis	#15a	Describe criteria under which study data will be quantitatively synthesised	11/12
Data synthesis	#15b	If data are appropriate for quantitative synthesis, describe planned summary measures, methods of handling data and methods of combining data from studies, including any planned exploration of consistency (such as I2, Kendall's τ)	11/12
Data synthesis	#15c	Describe any proposed additional analyses (such as sensitivity or subgroup analyses, meta-regression)	11/12
Data synthesis	#15d	If quantitative synthesis is not appropriate, describe the type of summary planned	NA
Meta-bias(es)	#16	Specify any planned assessment of meta-bias(es) (such as publication bias across studies, selective reporting within studies)	NA

For peer review only

Confidence in cumulative evidence	#17	Describe how the strength of the body of evidence will be assessed (such as GRADE)	NA

The PRISMA-P elaboration and explanation paper is distributed under the terms of the Creative Commons Attribution License CC-BY. This checklist was completed on 29. June 2022 using https://www.goodreports.org/, a tool made by the EQUATOR Network in collaboration with Penelope.ai

For peer review only
